# β-COP as a Component of Transport Vesicles for HDL Apolipoprotein-Mediated Cholesterol Exocytosis

**DOI:** 10.1371/journal.pone.0151767

**Published:** 2016-03-17

**Authors:** Weilie Ma, Margarita Lin, Hang Ding, Guorong Lin, Zhizhen Zhang

**Affiliations:** Department of Biochemistry and Molecular Biology, Key Laboratory of Medical Molecular Diagnostics of Guangdong Province, Guangdong Medical University, Dongguan, Guangdong, 523808, China; Wake Forest School of Medicine, UNITED STATES

## Abstract

**Objective:**

HDL and its apolipoproteins protect against atherosclerotic disease partly by removing excess cholesterol from macrophage foam cells. But the underlying mechanisms of cholesterol clearance are still not well defined. We investigated roles of vesicle trafficking of coatomer β-COP in delivering cholesterol to the cell surface during apoA-1 and apoE-mediated lipid efflux from fibroblasts and THP-1 macrophages.

**Methods:**

shRNA knockout, confocal and electron microscopy and biochemical analysis were used to investigate the roles of β-COP in apolipoprotein-mediated cholesterol efflux in fibroblasts and THP-1 macrophages.

**Results:**

We showed that β-COP knockdown by lentiviral shRNA resulted in reduced apoA-1-mediated cholesterol efflux, while increased cholesterol accumulation and formation of larger vesicles were observed in THP-1 macrophages by laser scanning confocal microscopy. Immunogold electron microscopy showed that β-COP appeared on the membrane protrusion complexes and colocalized with apoA-1 or apoE during cholesterol efflux. This was associated with releasing heterogeneous sizes of small particles into the culture media of THP-1 macrophage. Western blotting also showed that apoA-1 promotes β-COP translocation to the cell membrane and secretion into culture media, in which a total of 17 proteins were identified by proteomics. Moreover, β-COP exclusively associated with human plasma HDL fractions.

**Conclusion:**

ApoA-1 and apoE promoted transport vesicles consisting of β-COP and other candidate proteins to exocytose cholesterol, forming the protrusion complexes on cell surface, which were then released from the cell membrane as small particles to media.

## Introduction

High-density lipoproteins (HDL) carry approximately one third of the cholesterol in human blood. These particles also contain phospholipids and apolipoproteins, the major one being apoA-1 but including others such as apoE to a smaller extent. Population studies show that plasma HDL levels inversely correlate with the incidence and prevalence of cardiovascular disease [1, 2]. Approximately every milligram increase of HDL is estimated to reduce the mortality rates of cardiovascular disease by 2 to 4 percent [3]. This beneficial role is partially attributed to the ability of HDL, in particular its apolipoproteins that promote cholesterol efflux from foam cells, to reduce lipid accumulation and consequently decrease the risks of cardiovascular disease. In this context, how apolipoproteins promote removal of cellular cholesterol is not only a fundamental mechanism of cell biology but is also central to development of new treatment for atherosclerotic cardiovascular disease, a major cause of mortality worldwide.

HDL apolipoprotein-mediated cholesterol efflux pathway has been known to require a binding protein/receptor [4], signaling transduction [5], and Golgi and vesicle transport [6, 7], the latter which is sensitive to COP I vesicle inhibitor brefeldin A, a fungi metabolite. COPI vesicles consist of seven coatomer subunits (α, β, β', γ, δ, ε, ζ) and an ADP ribosylation factor (ARF). ARF is a GTP binding protein and is activated by exchange of GDP with GTP through guanyl-nucleotide exchange factors (GEF). The activated ARF then binds to β-COP subunit and recruits other coatomers to form transport vesicles. Brefeldin A binding to GEF isoforms BIG1 or BIG2 causes the disintegration of Golgi structure, blocks vesicle transport and reduces apoA-1 mediated cholesterol efflux. Expression of the dominant negative form of ARF or siRNA knockout of BIG1 also inhibits apoA-1-mediated cholesterol efflux [6–8] while increasing intracellular cholesterol accumulation [9]. However, whether β-COP itself participated in the apoA-1–mediated cholesterol efflux pathway has not been determined. Aim of this study was to investigate if β-COP was required in the cholesterol efflux pathway, by using combinations of biochemical analysis, confocal and electron microscopy as well as shRNA knockout in fibroblast and THP-1 macrophages. We reported here that β-COP was crucial for apolipoprotein-mediated cholesterol efflux pathway.

## Methods

### Ethics Statement

Use of human blood in this study conformed to the principles outlined in the Declaration of Helsinki. Written consents were obtained from healthy donors prior to blood samples in the study and the Ethical Committee of Guangdong Medical University approved the study.

### Cell Culture

Human monocytic leukemia cell line THP-1 was purchased from the ATCC. Cell culture and setup for individual experiments were identical to the procedures as described in our recent study [10]. Normal human skin fibroblasts and Tangier disease fibroblasts were maintained and prepared for experiments according to the methods in the literature [11].

### β-COP Specific Lentiviral shRNA and Transduction

The human β-COP specific shRNA oligonucleotide sequences were synthesized by Genomeditech Co., Ltd. (Shanghai, China), cloned into the pGMLV-SC1 RNAi lentiviral vector (Invitrogen Life technologies, Grand Island, NY, USA), and then subjected to sequence verification. HEK 293T cells were co-transfected with the β-COP-shRNA vector and Lenti-HG Mix using HG transgene reagent to generate the β-COP-shRNA lentiviral particles. THP-1 cells were transduced with the lentivirus at 30 MOI and expression of GFP protein level from the pGMLV-SC1 sequence under control of CMV promoter was used to monitor transduction efficiency. The sequences for construction of β-COP-shRNA vector were 5'gatccGCATTGCGCTATGTAGCTTTG TTCAAGAGA CAAAGCTACATAGCGCAATGCTTTTTTg3' (forward) and 5'aattc AAAAAAGCA TTGCGCTATGTAGCTTTGTCTCTTGAACAAAGCTACATAGCGCAATGCg3' (reverse) while the sequences for the negative control shRNA were 5'gatccGTTCTCCGAACGTGTCACGTTTCAAGAGAACGTGACACGTTCGGAGAA CTTTTTTACGCGTg3' (forward) and 5'aattcACGCGTAAAAAAGTTCTCCGAACGT GTCACGTTCTCTTGAAACGTGACACGTTCGGAGAACg3' (reverse).

### QRT-PCR Analysis

Total RNA was extracted using Trizol reagent (Life Technologies, Grand Island, NY USA) according to the manufacturer’s instructions. QRT-PCR was conducted in the ABI 7500 Real-Time PCR system (Applied Biosystems, Weiterstadt, Germany) with reagents obtained from TaKaRa Biotechnology Co., Ltd. (Dalian, China). Total RNA (300 ng) from each condition was used for the first strand synthesis. PCR cycles were performed at the conditions as following: 95°C for 30s, 95°C for 5s and 60°C for 34s with 40 cycles, 95°C for 15s and 60°C for 1min and 95°C for 15s with 1 cycles. The QPCR primers for β-COP were GCAACTCAGAGTGCCCTTAGCA (forward) and GCAATCTTGGTCAGAGTTGTGGC (reverse) while primers for GAPDH were GTCTCCTCTGACTTCAACAGCG (forward) and ACCACCC TGTTGCTGTAGCCAA (reverse).

### Cholesterol Efflux Assay

Cholesterol efflux assay was conducted as described in our recent study [10], with [^3^H] cholesterol at specific activity 10.5 Ci/mmol from the Shanghai Atomic Institute, Chinese Academy of Sciences (Shanghai, China). [^3^H] cholesterol in the media and cells was determined separately by using a scintillation counter. Cholesterol efflux (%) was calculated as the counting in the efflux media divided by total counting (media plus cells) and multiplied by 100%. Net apoA-1-mediated cholesterol efflux was obtained by subtracting the counting from control cells from that of the cells incubated with apoA-1.

### Confocal Microscopy

THP-1 macrophages transduced by β-COP shRNA lentivirus and controls were cultured on glass cover slips and were loaded with or without 50 μg/ml acLDL for 48 h. The resulting cells were treated with or without 10 μg/ml apoA-1for 6 hours, then fixed in 4% paraformaldehyde in PBS for 20 min, and permeabilized and blocked in 5% BSA in PBS containing 0.1% NP 40 for 45 min. After incubation with rabbit anti-ADFP antibody (Abcam) at 1:100 dilutions at 4°C overnight, cells were washed three times for 10 min each with 5% BSA in PBS containing 0.1% NP 40, incubated with goat anti-rabbit Alexa 568-conjugated secondary antibody (Abcam) at 1:1000 dilutions for 1 h at room temperature and washed again at the same conditions for three times. The cover slips were mounted with ProLong^®^ antifade reagents and were allowed to dry. Slides were examined with a confocal laser-scanning microscope (Zeiss LSM 700, Germany). Cell images were acquired with software Zen. Fiji (Image J) was used to determine fluorescence intensity in same square area with and without cells in arbitrary units. The relative fluorescence units (or intensity) were obtained by subtraction of the background fluorescence from the cell fluorescence.

### Electron Microscopy

Electron microscopy studies in fibroblasts and THP-1 macrophages were carried out as previously described by Lin and Oram [11]. Briefly, cholesterol-loaded cells were incubated with serum-free media containing 1mg/ml BSA alone or with the additions of 10 μg/ml apoA-I or apoE at 37°C for 6 hours. Cells then were quickly washed twice with PBS to remove unbound apolipoprotein and prefixed with 4% paraformaldehyde in PBS (pH 7.2) for 30 min to preserve cell morphology. Mouse anti-β-COP antibody (Sigma, 1:250 dilution) and goat anti-apoA-I antibody (Academy Bio-medical Co. 1:1000) were used for determining co-localization of β-COP and apoA-1 while mouse anti-β-COP antibody (Sigma, 1:250 dilution) and goat anti-apoE antibody (Academy Bio-medical Co. 1:1000) were used for determining co-localization of β-COP and apoE. Secondary antibodies were 18 nm colloidal gold of donkey anti-mouse IgG and 12 nm gold of donkey anti-goat IgG (Jackson ImmunoResearch Laboratories, Inc.). The specimens were examined with a transmission electron microscope (model 100, Philips Electron Optics USA) at an accelerating voltage of 60 kV.

### Western Blotting Analysis

To prepare samples for Western blotting, total cellular protein was extracted with RIPA buffer (Beyotime Institute of Biotechmolygy, Beijing China). Microsomal membranes were obtained by ultracentrifugation at 100,000 x g for 30 min after unbroken cells and nuclei were removed from total cell homogenates by centrifugation at 3000 x g twice for 10 min at 4°C. Western blotting was carried with standard protocol with anti-β-COP antibody (1:1000, clone MAD, Sigma), anti-GAPDH antibody, anti-ADFP antibody or anti-P4HB (1:1000 dilutions, Abcam). Secondary antibodies conjugated with HRP were used at 1:40000 dilutions (Jackson ImmunoResearch Laboratories, Inc). Protein bands were detected with ECL and quantified with Molecular Imager ChemiDoc XRS+ System (Bio-Rad, Wuhan, China).

To label the cell surface proteins, live cells were incubated with 10 μg/ml apoA-1 for 30 min at 37°C and were subsequently treated with 1mg/ml of non-permeable sulfo-NHS-biotin in PBS on ice for 30 min to biotinylate only the cell surface proteins. The cells were then solubilized with 1% Triton/PBS buffer containing protease inhibitors. The protein concentration was determined and aliquots were saved for Western blotting to determine total level of β-COP. Equal amount of the cell extracted protein from each condition was incubated with mouse anti-β-COP antibody (Sigma, 1:250 dilutions) and followed by anti-mouse IgG-coated magnetic beads (Dynal). The beads were collected by a magnet and then washed twice with Triton/PBS buffer. The biotinylated proteins were eluted from the beads for Western blotting analysis and detected by streptavidin-HRP and ECL.

To obtain secretory proteins from the conditioned cell culture media, the media were centrifuged at 1000 g for 10 min to pellet cell debris and the supernatant was transferred to a new tube. Proteins were precipitated from the supernatant by 10% TCA and pellets were recovered by centrifugation. The protein pellets were washed and were processed either for Western blotting or proteomic analysis.

### Proteomic Analysis of the Media Proteins

The proteins obtained from the media as described above were digested with trypsin (1 μg/50 μg protein, Promega) at 37°C overnight. The resulting peptides were purified with a stage tip [12], and re-suspended in 0.5% acetic acid for proteomics analysis, which was accomplished with Q Exactive mass spectrometer (Thermo Fisher Scientific) according to the methods from manufacturer [13]. The spectrum data obtained was searched against human protein database to identify target proteins.

### Detection of β-COP in Human Plasma and Lipoprotein Fractions

Lipoproteins were separated from 0.5 ml human plasma by FPLC according to the method described by Innis-Whitehouse and colleagues[14]. Cholesterol was determined in each fraction by using a cholesterol assay kit (Sigma) following the manufacturer’s protocol. Human plasma (5 μl) or equal volume (25μl) from each FPLC fraction was subjected to SDS PAGE and Western blotting as described above to detect β-COP with two different anti-β-COP monoclonal antibodies (Sigma, the clone MAD at 1:500 or the clone M3A5 at 1:125 dilutions).

### Determination of Total Cellular Cholesterol in Culture Cells

THP-1 macrophages were transduced with β-COP shRNA or scrambled shRNA lentivirus at 30 MOI as described above, along with non-transduction control. Total 1x 10^6^ cells were seeded to each well in 6-well dishes. The cells were induced by PMA and were then loaded with or without 50 μg/ml acLDL for 48 h as above. The resulting cells were treated with or without 10 μg/ml apoA-1 for 6 hours. The culture media were then removed and the cells were washed three times with PBS. The resulting cells were lysed and total cholesterol was assayed in duplicate with an assay kit (Applygen Technologies Inc. Beijing, China) according to the manufacturer’s instruction. Total cellular cholesterol was expressed as nmol/ mg protein (mean± SE of three separate experiments).

### Statistical Analysis

Statistical analysis was performed using one-way ANOVA with GraphPad Prism (GraphPad Software), followed by the Newman-Keuls multiple comparisons test. The level of significance was set at P < 0.05 for all results.

## Results

### Efflux Reduction and Cholesterol Accumulation in Larger Vesicles by β-COP Lentiviral shRNA Knockdown

**Fig 1:**
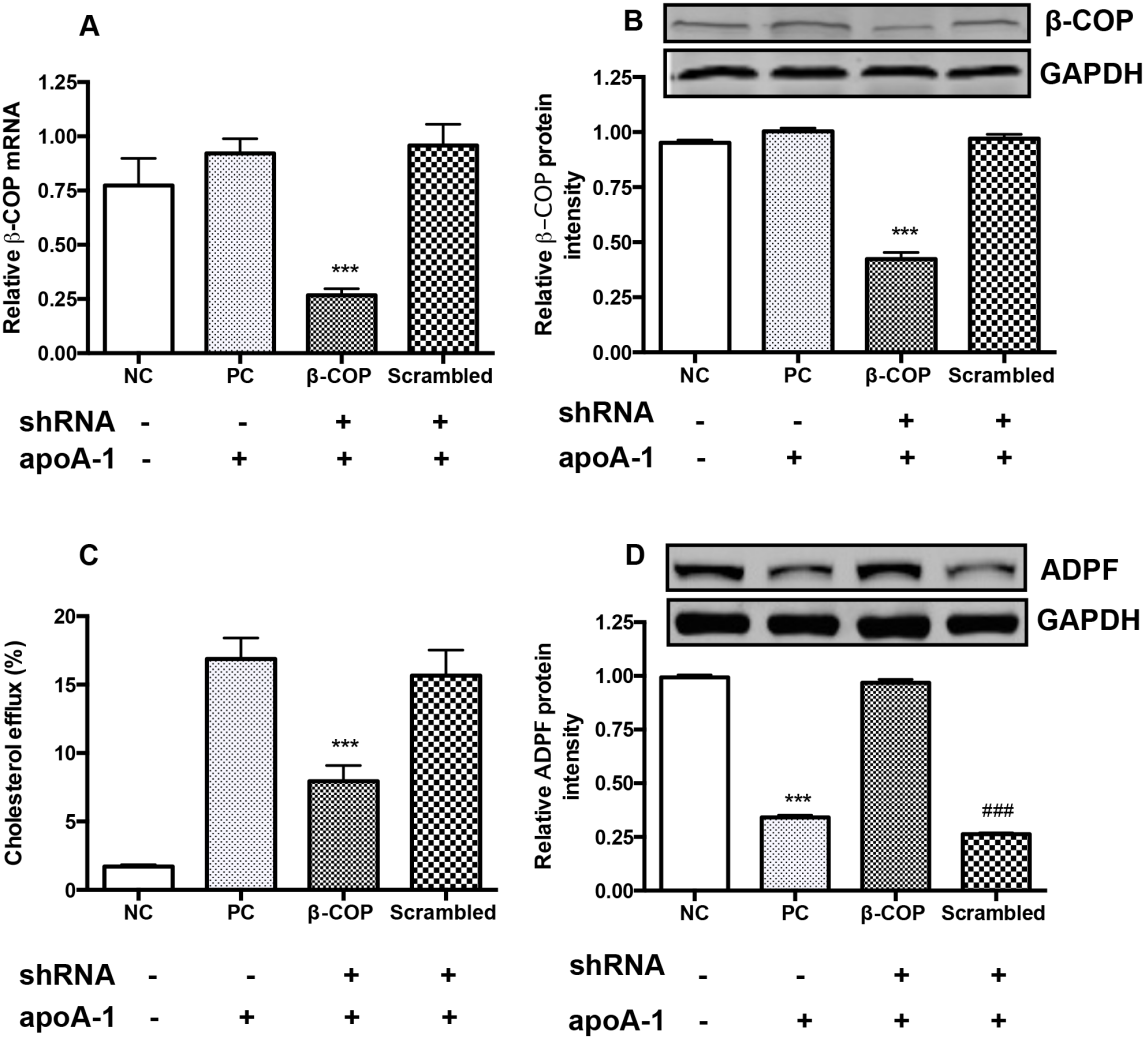
Effect of β-COP shRNA on the designated protein expression and cholesterol efflux of THP-1 macrophages. (A) β-COP mRNA levels determined by qRT-PCR. Experimental conditions are indicated in the x-axis labeling: NC refers to negative control, PC means positive control, and the β-COP and scrambled labels indicate the corresponding shRNA treatment. Minus signs indicate no shRNA transduced or apoA-1 incubation, while plus signs designate β-COP shRNA, scrambled shRNA or apoA-1 treatment. The same labels were also used in the entire study where applicable. ***, P<0.001, compared to all. (B) β-COP protein levels determined by Western blotting. ***, P<0.001, compared to all. (C) ApoA-1 mediated cholesterol efflux. The cells were simultaneously loaded with 50 μg/ml of acetylated LDL and 0.2 μCi [^3^H] cholesterol. ApoA-1 mediated [^3^H] cholesterol efflux was determined as Method. ***, P<0.001, compared to the PC and scrambled shRNA transduced groups. (D) ADPF protein levels determined by Western blotting. ***, P<0.001, compared to the NC and β-COP shRNA transduced groups. ###, P<0.001, compared to the NC and β-COP shRNA transduced group. Data were the mean of five repeated experiments ± SEM (n = 5).

In comparison to control, the levels of β-COP mRNA and protein were decreased in the β-COP-shRNA lentivirus transduced THP-1 macrophages by 74.0% and 76.4%, respectively (Fig 1A and 1B). Functionally, β-COP knockdown reduced apoA-1-mediated cholesterol efflux by more than 50% in the macrophages whereas the cholesterol efflux was not affected in the non-transduced control cells or the scrambled shRNA lentivirus transduced cells (Fig 1C). Adipophilin (ADFP), a lipid droplet coat protein, is located on the outermost monolayer of lipid droplets in THP-1 macrophages [15] and has been used as a specific marker of lipid accumulation in THP-1 macrophages and atherosclerotic lesions [16, 17]. Consistent with these previous reports, western blotting showed that ADFP levels were reduced in the non β-COP shRNA-transduced cells or scrambled lentivirus transduced cells following incubation with apoA-1, while ADFP levels remained high in control cells without incubation with apoA-1 as well as the cells transduced with β-COP shRNA lentivirus and followed incubation with apoA-1 (Fig 1D).

**Fig 2:**
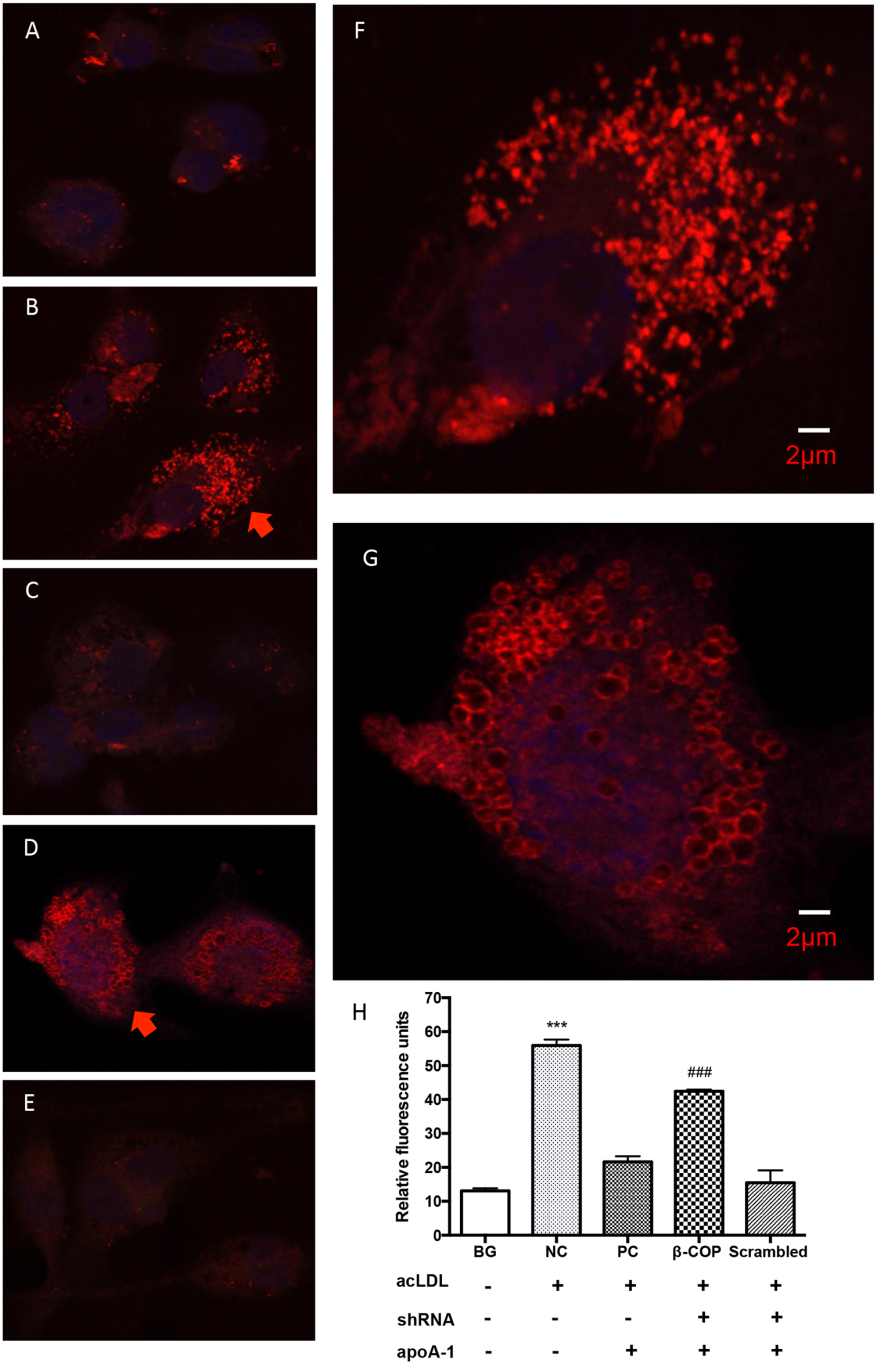
Cellular cholesterol localization with ADFP as an indicator in β-COP shRNA transduced macrophages observed by a confocal laser scanning microscopy. Typical images from each group are shown here. Additional cell images are available in the Support Information Files. (A) Non cholesterol-loading THP-1 macrophages, showing background fluorescence. Relative fluorescence units measured are shown in Fig 2H, BG (background) column. (B) THP-1 macrophages loaded with cholesterol, showing intense punctate fluorescence. A closer view of the cell indicated by the arrow is shown in Fig 2F. Higher fluorescence intensity is also shown in Fig 2H, NC column. (C) THP-1 macrophages loaded with cholesterol and subsequently incubated with apoA-1, with nearly invisible fluorescence and a low level of quantification in Fig 2H, PC column. (D) THP-1 macrophages transduced with β-COP shRNA, loaded with cholesterol and then treated with apoA-1. Vesicles with intense fluorescence are clearly visible. A closer view of the cell indicated by an arrow is shown in Fig 2G, displaying vesicles in size from 500 to 1000 nm in diameter. High fluorescence levels are also reflected in Fig 2H, the β-COP column. (E) THP-1 macrophages transduced by the scrambled shRNA, loaded with cholesterol and then treated with apoA-1, no visible vesicles and also low in fluorescence in Fig 2H, the scrambled column. (H) Relative fluorescence intensity in each condition. *** and ###, P<0.001, compared to BG, PC and scrambled groups. Error bars represent mean ± SEM from 80–100 cells (16 to 20 cells from each experiment, repeated five times).

To observe cellular cholesterol distribution in macrophages under the conditions above, a confocal laser scanning microscopy was used with ADFP as an indicator. Cell fluorescence was found to be dramatically increased in dense punctate distribution in acLDL-loaded macrophages (Fig 2B and 2F), in comparison to non-acLDL-loaded cells (Fig 2A). The fluorescence became nearly absent in the non-transduced cells followed by apoA-1 incubation (Fig 2C) or in scrambled lentivirus transduced cells with apoA-1 incubation (Fig 2E) because apoA-1 promoted cholesterol efflux from the cells and reduced intracellular cholesterol under the condition (Fig 1C). The fluorescence remained high and was mainly associated with the vesicles (usually from 500 to 1000 nm, some up to 2000 nm in diameters) in the β-COP shRNA silenced macrophages (Fig 2D and 2G). The relative fluorescence units, indications of cellular cholesterol levels, also reflected these changes by quantitation of the red fluorescence in each condition (Fig 2H). Thus, the results show β-COP was an important component of cholesterol transport vesicles for apoA-1-mediated cholesterol efflux pathway.

In order to provide additional evidence that β-COP shRNA knockout leaded to cellular cholesterol accumulation, the total intracellular cholesterol was also determined. Results showed that the mean total cholesterol levels in three separate experiments were 50±8.0, 90.3±8.4, 57. 9±2.4, 114±18.24 and 48.9±8.4 nmol cholesterol/mg protein in BG, NC, PC, β-COP shRNA and scrambled shRNA groups, respectively. Statistical analysis showed that cholesterol levels were significantly higher in NC and β-COP shRNA transduction groups (P <0.001, compared to BG, PC and scrambled groups). Thus, data of the ADFP expression and direct measurement of cholesterol were consistent, showing that β-COP was crucial for apoA-1-mediated vesicle transport of cholesterol removal from cells.

### β-COP on the Cell Surface Observed by Electron Microscopy

**Fig 3:**
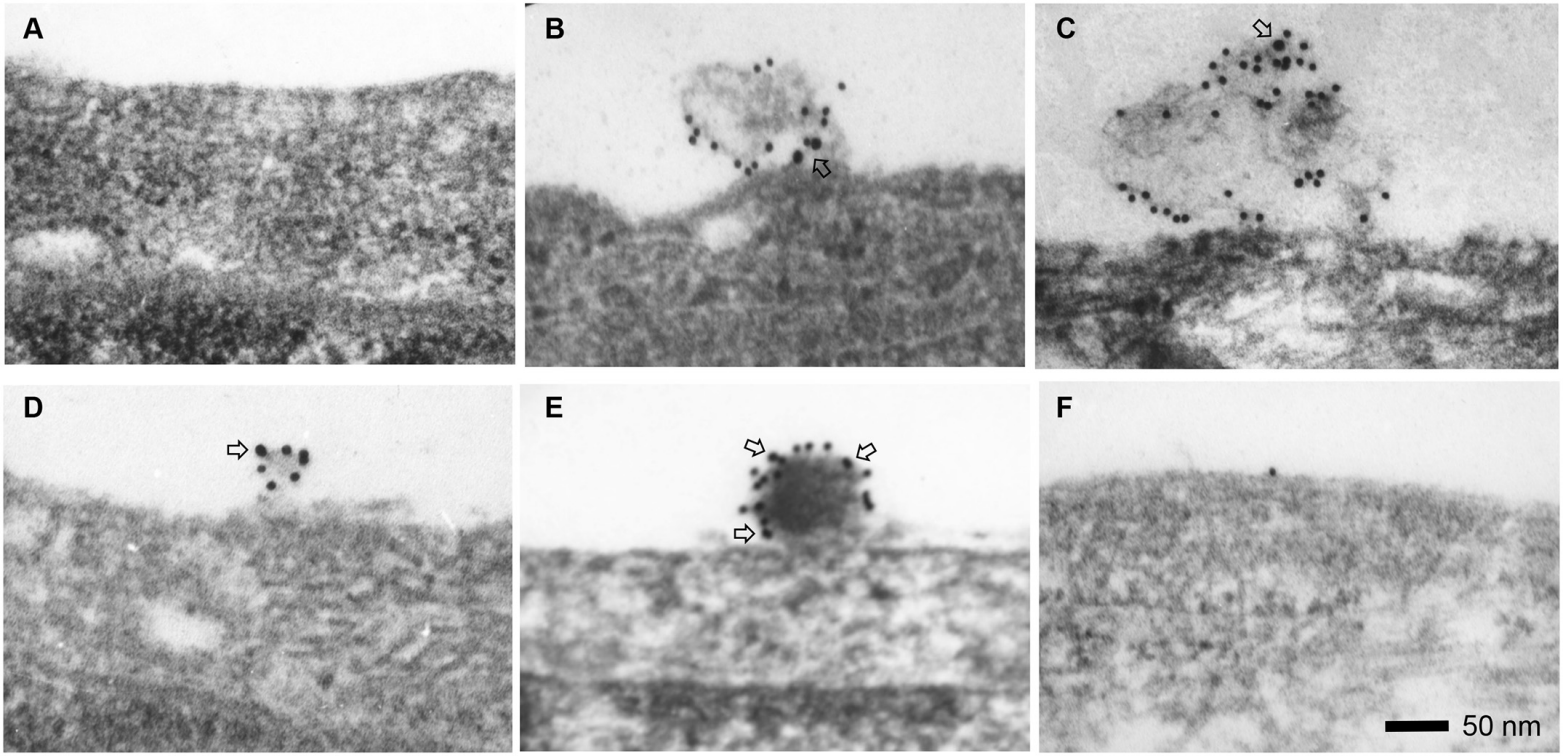
Colocalization of β-COP and apoA-1 or apoE on the cell surface observed by immunogold electron microscopy. β-COP is indicated by large gold particles (18 nm, open arrows) while apoA-1 and apoE are represented by small gold particles (12 nm). (A) No detection of β-COP on fibroblast without addition of apoA-1 to the culture medium during incubation. (B) ApoA-1 and β-COP colocalization on protrusion complex on the fibroblast surface with incubation of 10 μg/ml apoA-1 in the medium. (C) ApoE and β-COP colocolization on the fibroblast surface following incubation of 10 μg/ml apoE. (D) ApoA-1 and β-COP colocalization on protrusion complex on the THP-1 macrophage surface with incubation of 10 μg/ml apoA-1. (E) ApoE and β-COP colocalization on protrusion complex on THP-1 macrophage surface following incubation of 10 μg/ml apoE. (F) No protrusion complex on cell surface on two different Tangier fibroblast cell lines after treatment with apoA-1 or apoE. The experiments were repeated four times.

Previous studies show that apoA-1 mediated cholesterol efflux pathway is associated with formation of lipid complexes originally described as “mushroom” shaped protrusions between 10 to 200 nm in size on the plasma membrane of fibroblasts and THP-1 macrophages, but no cellular protein has been identified to associate within these membrane morphologies [11]. We hypothesized that β-COP might be a component of the membrane-bound lipid transport vesicles and we performed immunogold electron microscopy to investigate if β-COP would appear on “mushroom” shaped protrusions on the cell membrane. Results showed no detectable β-COP or evidence of lipid accumulation on the surface of cholesterol-loaded fibroblasts when no apoA-1 or apoE was added to incubate with cells (Fig 3A). With apolipoproteins added, β-COP was colocalized with apoA-1 or apoE on mushroom-shape protrusion in size from 100 to 200 nm on the cell surface in cholesterol-loaded fibroblasts (Fig 3B and 3C), and in relatively smaller sizes from 50 to 100 nm in cholesterol-loaded macrophages (Fig 3D and 3E), which were consistent with previous morphological observations [11]. In Tangier fibroblasts with defective ABCA1 and apoA-1 mediated cholesterol efflux pathway, there was no β-COP appearance on the cell surface (Fig 3F). There were no gold particles found inside of the cells, suggesting that apoA-1 or apoE did not enter cells under our experiment conditions.

When cells are incubated with apoA-1 or over expression of apoE, heterogeneous sizes of particles are found in culture media [18–20]. Similarly, we also observed various sizes of small particles by transmission electron microscopy in the culture media of THP-1 macrophage incubated with apoA-1 or apoE. Taken together, our results suggested that apoA-1 and apo E promoted the β-COP containing vesicles to transport cholesterol and exocytose to the cell surface. The cholesterol formed protrusion complexes and then subsequently was released as small particles into the media.

### Biochemical Studies of β-COP on the Cell Surface and Culture Media

**Fig 4:**
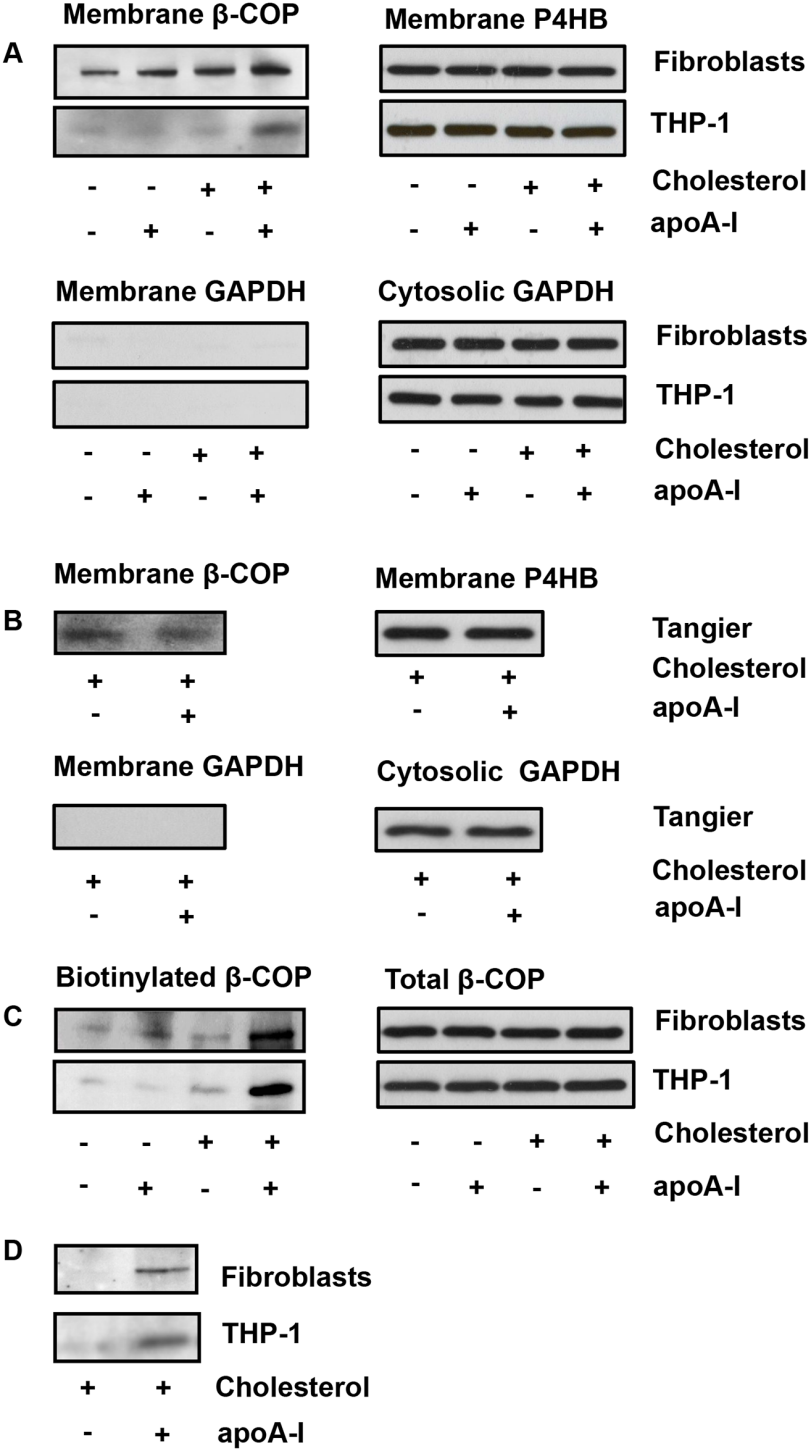
Translocation of β-COP to the cell membrane induced by apoA-1. (A) Increased β-COP on the cell membrane by apoA-1 is shown by Western blotting (left top panel). Membrane P4HB, due to its small molecular weight, served as a loading control in the same blotting (right upper panel). No detectable cytosolic GAPDH marker in the membrane (left lower panel), while it is abundant in the cytosolic fraction (right lower panel). (B) No β-COP translocation to the cell membrane on Tangier fibroblasts treated by apoA-1. Membrane marker P4HB is equal in each lane (top right panel) and no detection of cytosolic protein marker GAPDH in the membrane fraction (bottom left panel). Two Tangier fibroblasts cell lines from different patients were used (biological replicates = 2). (C) Increased β-COP translocation to the membrane by apoA-1 in the live cell surface-labeled assay (left panel). Total β-COP is equal in all lysate samples, indicating no alteration of β-COP expression and turnover by apoA-1 incubation. Total β-COP level also served as a control for equal amount protein used in immunoprecipitation (right panel). (D) β-COP appeared on the culture media. β-COP in the media was detected by immunoblot analysis. Minus signs indicated no treatment, while plus signs indicated cholesterol loading or apoA-1 incubation. The experiments were repeated four times.

To confirm apoA-1-promoting β-COP appearance on the cell surface by independent methods, Western blotting was performed with the membrane proteins isolated from cell with and without apoA-1 treatment. Results showed that levels of β-COP on the cell membranes in response to apoA-1 were increased in both cholesterol-loaded normal fibroblasts and macrophages, with 2 to 3-fold increase seen in macrophages over control (Fig 4A, left upper panel) while membrane marker P4HP as a loading control was equal in each lane (Fig 4A, right upper panel). No cytosolic protein marker GAPDH was found in the cellular membrane (Fig 4A, left lower panel) although it was abundant in cytosolic fraction (Fig 4A, right lower panel). There was no change of the membrane β-COP level in non-cholesterol-loaded normal cells, or cholesterol-loaded fibroblasts from Tangier disease (Fig 4A and 4B, left upper panels).

To further unequivocally show translocation of β-COP to the cell membrane in response to apoA-1, the surface protein labeling was performed with non-permeable sulfo-NHS-biotin and the protein obtained by β-COP immunoprecipition was analyzed by Western blotting. Results showed that apoA-1 dramatically increased levels of β-COP on the cell surface in both cholesterol-loaded fibroblasts and macrophages cells, with β-COP almost absent on the cell surface in non-cholesterol-loaded cells (Fig 4C, left). Total cellular β-COP levels did not change under these conditions (Fig 4C, right), indicating that apoA-1 did not affect β-COP expression or prolong its turnover. β-COP was further detected in the culture media from cholesterol-loaded fibroblasts and macrophages incubated with apoA-1, but not in the control without apoA-1 (Fig 4D). Thus, results from biochemical analysis also supported the idea that apoA-1 promoted exocytosis of β-COP to the cell surface and secretion into the media.

### Proteomic Analysis of apoA-1 Conditioned Media

To identify if other proteins were released from the cells during cholesterol removal, proteomic analysis was performed with the samples precipitated with TCA from the apoA-1 conditioned cell culture media. Seventeen additional proteins were found in the apoA-1 conditioned media from THP-1 macrophages (Table 1), but absent in the control media without apoA-1. These proteins belonged to groups of coatomers (α-COP and β-COP), cell signaling, vesicle transport, and microtubule cytoskeleton and heat shock proteins. Among them, six proteins have been previously identified to associate with HDL particles by proteomic analysis[21]. It was also worth mentioning that both Western blotting and proteomic analysis identified presence of β-COP in the apoA-1 conditioned media.

**Table 1:** Proteins identified in apoA-1 conditioned media by proteomics.

ID	Unique Peptides	Protein name	MW (Da)	Coverage(%)
ACTA_human	11	α actin 2*	42,009	58.56
ACTB_human	13	β actin*	41,737	85.10
AMPN_human	5	Aminopeptidase N*	109,412	8.2
ANXA6_human	10	Annexin A6	75,873	29.73
BGH3_human	10	Transforming growth factor-beta-induced protein ig-h3	74,681	57.04
CAP1_human	9	Adenylyl cyclase-associated protein 1	51,901	49.02
CLH1_human	26	Clathrin heavy chain 1	191,615	28.8
COPA_human	9	α-COP	13,8346	11.80
COPB_human	9	β-COP	102,487	15.00
ENOA_human	10	α-enolase	47,169	45.79
GELS_human	10	Gelsolin*	85,697	12.83
HSBP1_human	3	Heat shock protein β1	22,783	52.14
HSP7C_human	3	Heat shock cognate 71 kDa protein	53,518	15.02
HS9A_human	6	Heat shock protein HSP 90α	84,660	13.60
HS9B_human	7	Heat shock protein HSP 90β	83,264	15.70
LAMP1_human	6	Lysosome-associated membrane glycoprotein 1	44,882	20.05
TBA1_human	5	Tubulin α chain*	50,327	22.41
TBB1_human	6	Tubulin β1 chain*	50,327	36.06

The proteomics analysis was repeated three times, and only entries reproducible each time were listed above.

*, symbol indicates that the proteins have been reported as HDL associated proteins (reference 21).

### β-COP Appearance in Plasma HDL

**Fig 5:**
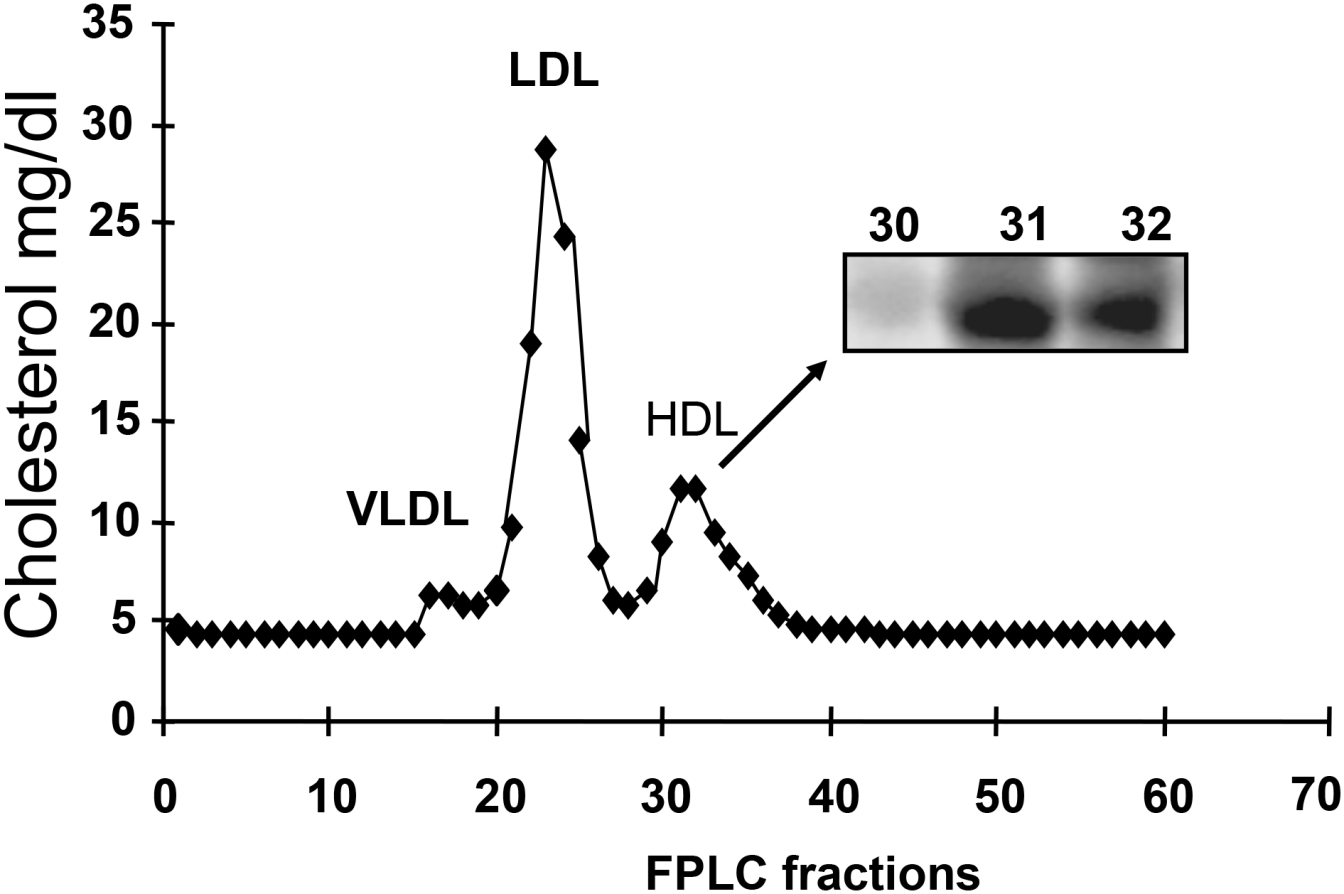
Plasma HDL fractions associated with β-COP. Lipoprotein fractions from normal human serum were isolated by FPLC, and equal volumes from each FPLC fraction were subjected to SDS PAGE and assayed for β-COP by Western blotting analysis. The β-COP positive fractions were shown in the inset. The Western blots were repeated three times using plasma samples from three healthy donors (biological replicates = 3).

To investigate if β-COP was detectible in human plasma, Western blotting was performed with lipoprotein after plasma lipoproteins VLDL, LDL, and HDL were isolated according to their particle size by FPLC. β-COP appeared exclusively in the HDL fractions (31 and 32) as determined by Western blotting (Fig 5). No β-COP was detected in the rest of fractions. Identical results were obtained with two different monoclonal antibodies (results not shown). The results suggested that secretion of β-COP and cholesterol from cells might be processed into a part of HDL *in vivo*.

## Discussions

The homeostasis of intracellular cholesterol pool is regulated through cholesterol synthesis, lipid entry through LDL receptor-mediated endocytosis and HDL apolipoprotein-mediated cholesterol efflux in majority of cells. Because macrophages take up oxidized and other modified LDL by a scavenger receptor-mediated endocytosis that is not subjected to a negative feedback regulation [22], HDL apolipoprotein-mediated cholesterol efflux becomes a crucial pathway in the prevention of lipid accumulation and atherogenesis. This study shows β-COP acting as an important component of the vesicles that transport and exocytose cholesterol to cell surface, forming lipid complexes in response to HDL apolipoproteins.

Apolipoprotein A-1 and apoE promote a time, energy and Golgi-dependent formation of lipid complexes on the cell membrane, which are originally described as a “mushroom” shaped protrusions 10 to 200 nm in size on the plasma membrane of fibroblasts and THP-1 macrophages and no lipid accumulation was observed on the cell membrane in the fibroblasts from Tangier disease incubated with apoA-1 or apoE [11]. Similar observations are later reported in aortic endothelial cells and J774 mouse macrophages [23, 24], suggesting that this is a common mechanism in different cells to eliminate excess cholesterol. In this study, we provide several lines of evidence to support β-COP as a part of the cholesterol transport vesicles in the apoA-mediated cholesterol efflux pathway. First, shRNA knockout of β-COP blocks apoA-1-mediated cholesterol efflux and leads to cholesterol accumulation in a larger vesicles. Secondly, immunogold microscopy shows colocalization β-COP with apoA-1 and apoE on the mushroom-shape protrusion complexes on the cell surface in both fibroblasts and THP-1 macrophages, but not in Tangier fibroblasts as described in the previous study [11]. Third, live cells labeled by non-permeable sulfo-NHS-biotin shows that β-COP is translocated to cell membrane in response to cholesterol loading and apoA-1 stimulation. Fourth, secretion of β-COP is detected only in the apoA-1 conditioned media by both Western blotting and proteomic. All these evidences support that β-COP acts as an important components of the transport vesicles for exocytosis of cholesterol into media.

ABCA1 mutations cause a severe HDL deficiency syndrome, cholesterol accumulation in tissue macrophages and atherosclerosis in Tangier disease [25–27]. ABCA1 is proposed to directly transport cholesterol and phospholipids across the cell membrane since it shares similar amino acid sequence with other ABC transporters [23, 28, 29]. However, recent studies show that ABCA1 neither binds nor transports cholesterol across the membrane, and its ATPase activity is surprisingly inhibited by a higher concentration of cholesterol [30, 31]. By using GFP tagged ABCA1, the protein is found to shuttle rapidly in the vesicles between the cell surface and intracellular compartments, which is sensitive to brefeldin A inhibition, indicating that ABCA1 plays role in vesicle transport rather than an active transporter across the plasma membrane.

All components in COPI vesicle are required for neutral lipid transport, as knockdown of any COPI subunit or ARF by siRNA leads to intracellular lipid accumulation in larger droplets[32–34], showing that COPI vesicles are critical for cellular transport and storage of neutral lipids in the droplets. Brefeldin A blocks apolipoprotein–mediated lipid efflux [6, 7], and inhibits the release of cellular lipids to form the protrusion on the cell surface [11]. Immunoprecipitation with anti-BIG1 antibody co-precipitates ABCA1 from liver vesicle membrane [9], suggesting that BIG1 co-locates within the same vesicles with ABCA1. BIG1 siRNA silence reduces the presence of ABCA1 in the cell membrane and cholesterol efflux to media while increased accumulation of intracellular cholesterol [9]. Taken together, these findings indicate that β-COP, ARF, BIG1, ADFP and ABCA1 are all components of the transport vesicles for the apoA-1-mediated cholesterol secretory pathway. When apoA-1 and apoE bind to a receptor on the cell membrane, it promotes these vesicles to transport and exocytose cholesterol to the cell surface forming protrusion complexes. These lipid complexes are then released into media in form of small particles to the media, as previously proposed in the receptor-mediated exocytosis model [35].

In addition to β-COP, proteomic analysis finds additional 16 proteins in the apoA-1 conditioned media from THP-1 macrophage media. Of them, α-actin 2 (α smooth muscle actin) and β-actin are known for vesicle transport and they potentially guide the vesicles moving toward a particular location on the cell membrane [36]. The α tubulin, β tubulin and clathrin are previously identified as components of coat vesicles [37], and α tubulin has been shown to participate apoA-1 mediated cholesterol efflux in astrocytes [38]. Adenylyl cyclase-associated protein 1 is a signaling protein that regulates actin cytoskeleton function [39]. α-COP, β-COP and clathrin heavy chain 1 are vesicle coat proteins. Heat shock proteins HSPβ1, HSP71, HSP 90α and HSP 90β are molecular chaperones with broad cellular functions, including regulation of intracellular vesicle transport through ATP-dependent mechanisms [40, 41]. Annexin A6 participates in vesicle transport of intracellular cholesterol [42], and lysosome-associated membrane glycoprotein 1 contributes to cholesterol transport between two cells from macrophages to vascular smooth muscle cells [43]. Potential roles of α-enolase, aminopetidase N and gelsolin in cholesterol efflux pathway remain unknown. It has been reported that more than 200 unique proteins are associated with HDL particles as determined by proteomic analysis [21]. We have showed that β-COP is associated with HDL fraction and six proteins identified above in the culture media are also among HDL associated proteins [21] (Table 1). However, it remains unknown about functions of these proteins identified. We will investigate if these proteins, in particular α-COP, are components of the cholesterol transport vesicles in our ongoing studies.

Exosomes are small vesicles with diameter from 30 to 150 nm, which were first discovered in cultured cell lines [44], and then in differentiated reticulocytes [45, 46]. Now many cells are found to spontaneously release exosomes into culture media and body fluids. More than 4500 different proteins, RNA and other molecules have been identified within exosomes. These small vesicles are associated with diversified cellular functions, including elimination of obsolete cholesterol, phospholipids and proteins, and delivery of signaling mediators to other cells [47, 48]. Consistent with previous observations that small particles are released into the culture media when cells are incubated with apoA-1 or expression of apoE [18–20], we also find mainly heterogeneous sizes of small particles in the apoA-1 conditioned media. Thus, mechanisms involved in apolipoprotein-mediated cholesterol efflux pathway are different from those of exosome formation.

In summary, this study demonstrates a cellular mechanism that HDL apolipoproteins promote the β-COP containing vesicles’ transport of the intracellular cholesterol and exocytosis to the cell surface. Defect of this pathway in fibroblasts from Tangier disease, which is associated with massive cholesterol accumulation in macrophages, implicates this pathway functions as a major mechanism to remove excess intracellular cholesterol and to protect against atherosclerosis.

## Supporting Information

S1 Fig An image of the background (BG) shows background fluorescence in non cholesterol-loaded THP-1 macrophage. The blue channel shows nuclei staining with DAPI. The green channel indicates expression of the GFP in shRNA construct or trace amount of autofluorescence as indicated. The red channel shows fluorescence from immunostaining of ADFP, an indicator of cholesterol level. The last channel shows merger of the three channels. The red fluorescence indicating for the cellular cholesterol is low.
(TIF)

S2 Fig Another image of the background (BG) fluorescence in non cholesterol-loaded THP-1 macrophage. (TIF)

S3 Fig Additional image of the background (BG) fluorescence in non cholesterol-loaded THP-1 macrophage. (TIF)

S4 Fig An image of the negative control (NC) shows that THP-1 macrophage loaded with cholesterol with intense punctate fluorescence in the red channel, indication of intracellular cholesterol accumulation. A trace amount of auto fluorescence is visible in the green channel.
(TIF)

S5 Fig An image of the negative control (NC) shows intense punctate fluorescence in the red channel in multiple cholesterol-loaded THP-1 macrophages. (TIF)

S6 Fig An image of the negative control (NC) shows intense punctate fluorescence in the red channel in cholesterol-loaded macrophages. (TIF)

S7 Fig An image of the positive control (PC) shows macrophages loaded with cholesterol and subsequently incubated with apoA-1, with nearly invisible fluorescence in the red channel. This indicates that apoA-1 promotes cholesterol efflux from the macrophages.
(TIF)

S8 Fig An image of the positive control (PC) shows macrophages loaded with cholesterol and subsequently incubated with apoA-1, as the S7 Fig. (TIF)

S9 Fig An image of the positive control (PC) shows macrophages loaded with cholesterol and subsequently with apoA-1, as the S7 and S8 Figs. (TIF)

S10 Fig An image of THP-1 macrophages transduced with β-COP shRNA, loaded with cholesterol and then treated with apoA-1. Vesicles with intense fluorescence are clearly visible and some vesicles are arranged in beautiful rosettes, supporting the concept that reduction of β-COP prevents cholesterol removal from the cells by apoA-1 mediated exocytotic pathway and leads to the cholesterol accumulation in the vesicles. The green channel also shows increased expression of the GFP from the β-COP shRNA lentivirus.
(TIF)

S11 Fig Images of THP-1 macrophages transduced with β-COP shRNA, loaded with cholesterol and then treated with apoA-1. Vesicles with intense fluorescence are clearly visible as the S10 Fig.
(TIF)

S12 Fig An image of THP-1 macrophages transduced with β-COP shRNA, loaded with cholesterol and then treated with apoA-1. Vesicles with intense fluorescence are clearly visible as the S10 and S11 Figs.
(TIF)

S13 Fig Images of THP-1 macrophages transduced by the scrambled shRNA, loaded with cholesterol and then treated with apoA-1. Red fluorescence is very low and the green fluorescence is visible, indicating that active expression of scrambled shRNA takes place in these cells but has minimal effect on apoA-1 mediated cholesterol efflux from the macrophages.
(TIF)

S14 Fig Images of THP-1 macrophages transduced by the scrambled shRNA, loaded with cholesterol and then treated with apoA-1. The cells have low red fluorescence as the S13 Fig.
(TIF)

S15 Fig Images of THP-1 macrophages transduced by the scrambled shRNA, loaded with cholesterol and then treated with apoA-1. The cells have low red fluorescence as the S13 Fig and Fig 14.
(TIF)
