# Supplementary figures and images for: β-COP as a Component of Transport Vesicles for HDL Apolipoprotein-Mediated Cholesterol Exocytosis
Source: PLoS One. 2016 Mar 17;11(3):e0151767. doi: 10.1371/journal.pone.0151767 (PMC4795675; doi:10.1371/journal.pone.0151767)

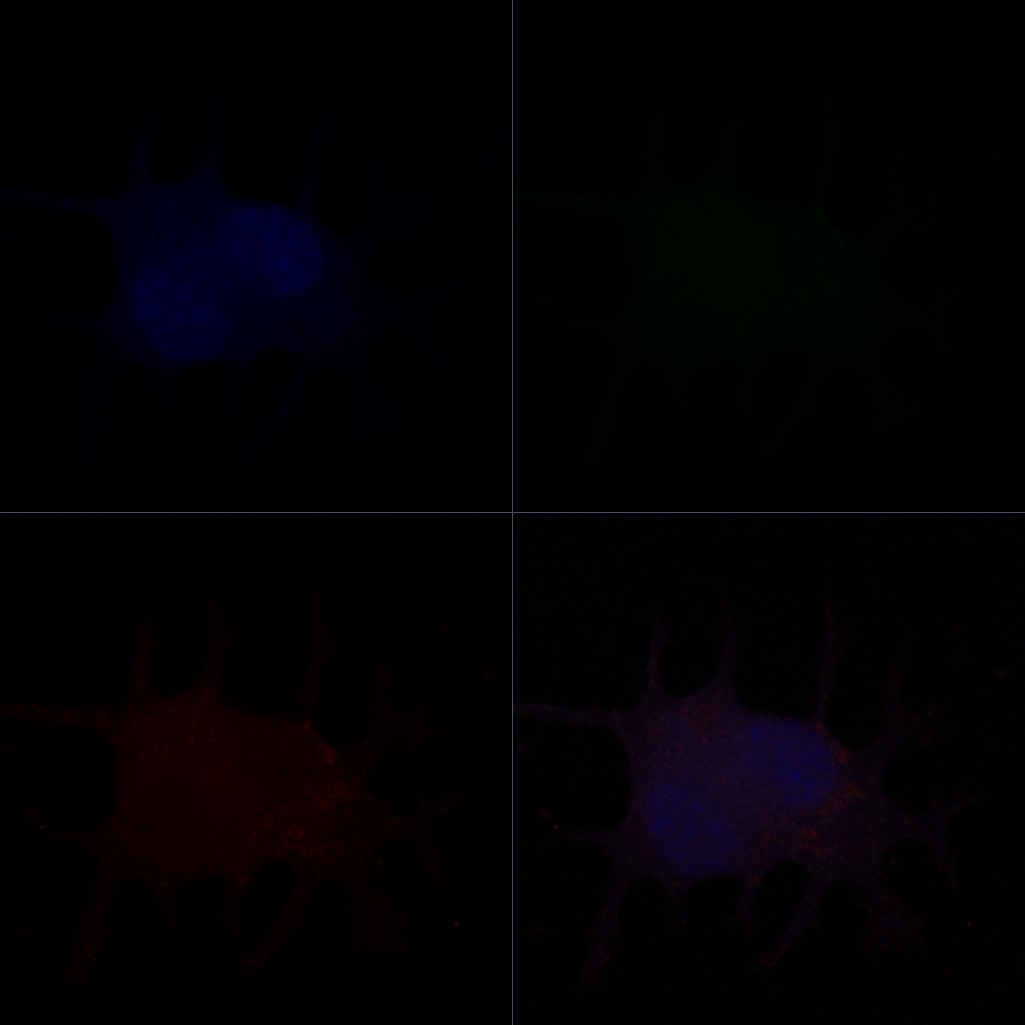

Supplement: S1 Fig — The blue channel shows nuclei staining with DAPI. The green channel indicates expression of the GFP in shRNA construct or trace amount of autofluorescence as indicated. The red channel shows fluorescence from immunostaining of ADFP, an indicator of cholesterol level. The last channel shows merger of the three channels. The red fluorescence indicating for the cellular cholesterol is low. (TIF) [file pone.0151767.s001.tif]

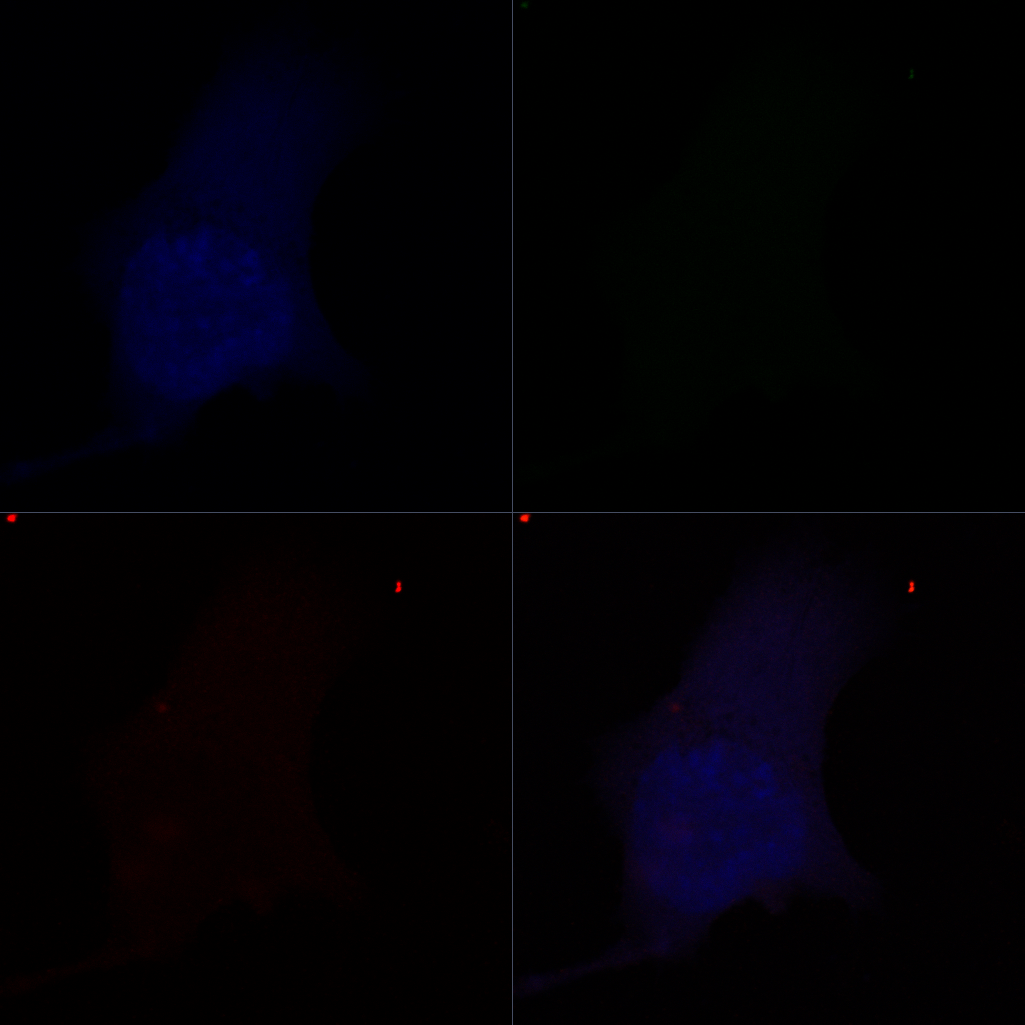

Supplement: S2 Fig — (TIF) [file pone.0151767.s002.tif]

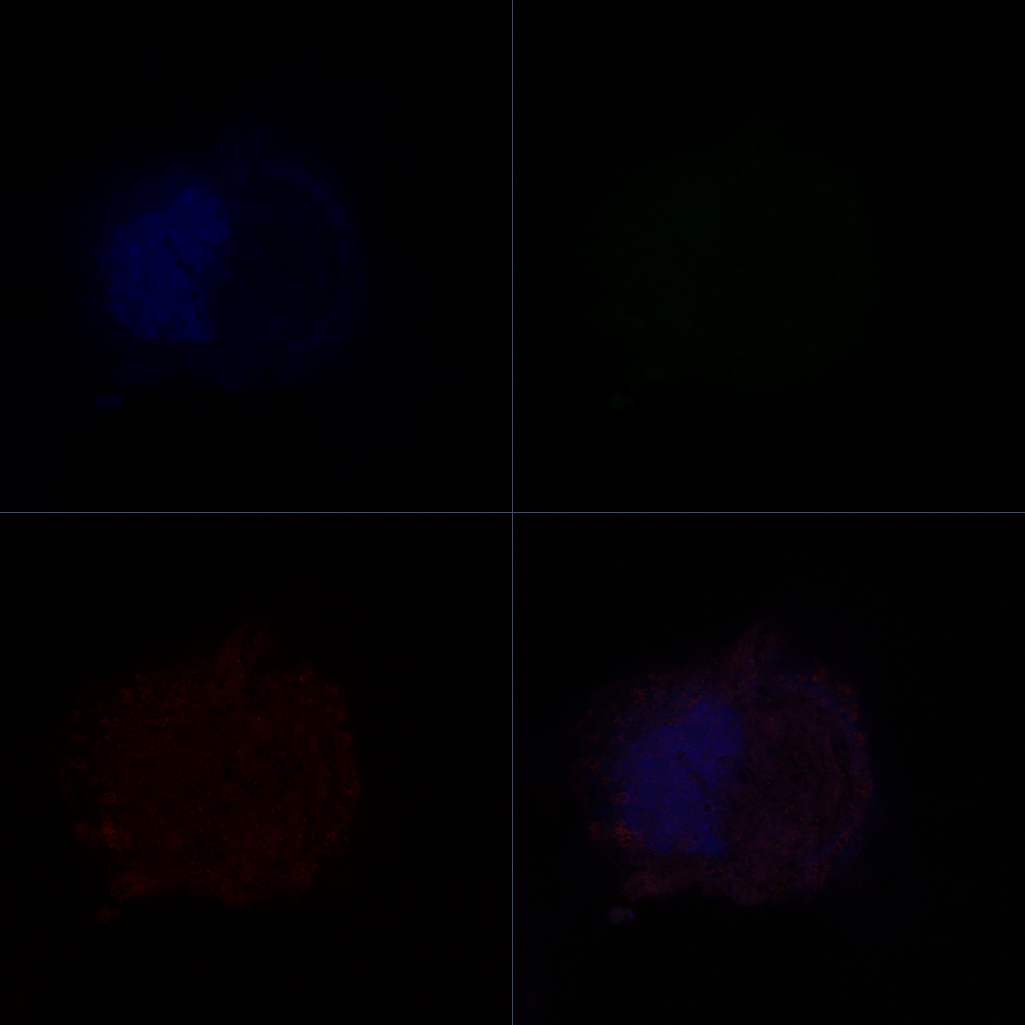

Supplement: S3 Fig — (TIF) [file pone.0151767.s003.tif]

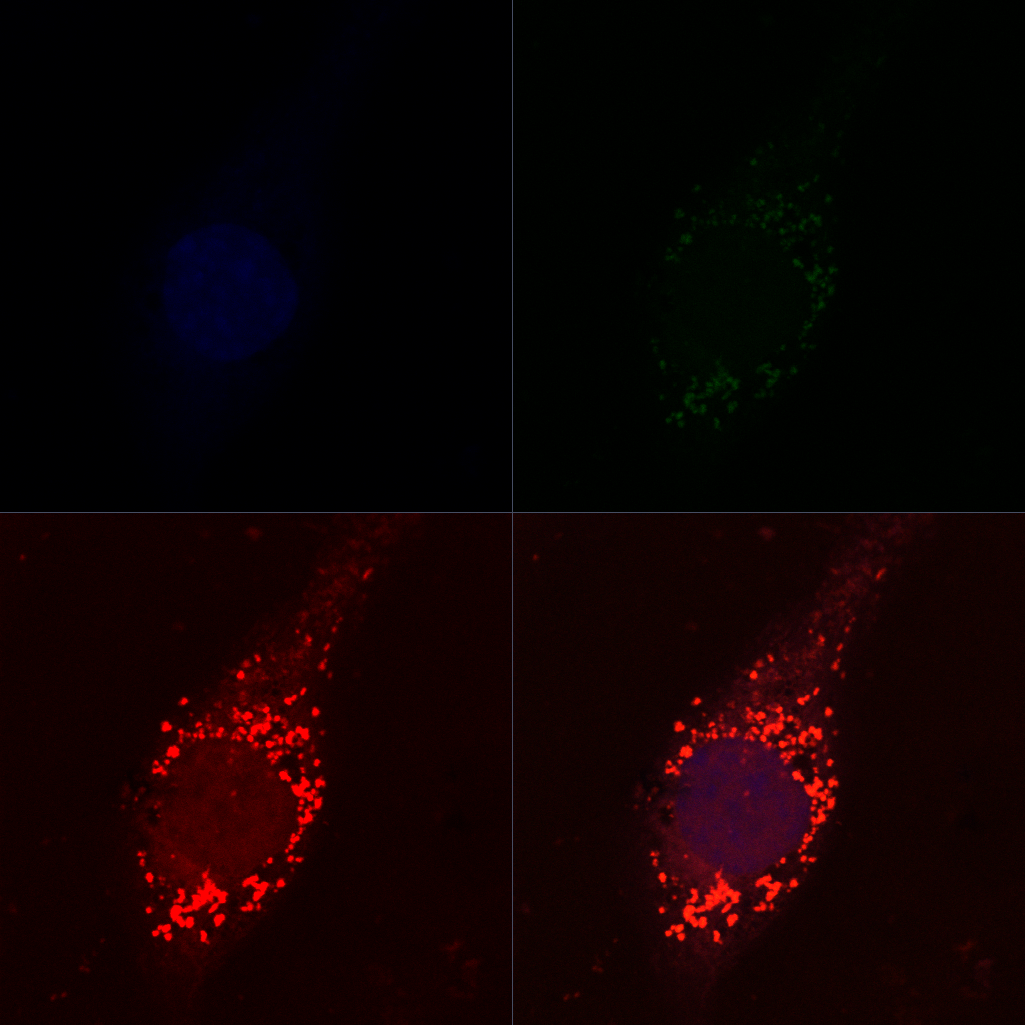

Supplement: S4 Fig — A trace amount of auto fluorescence is visible in the green channel. (TIF) [file pone.0151767.s004.tif]

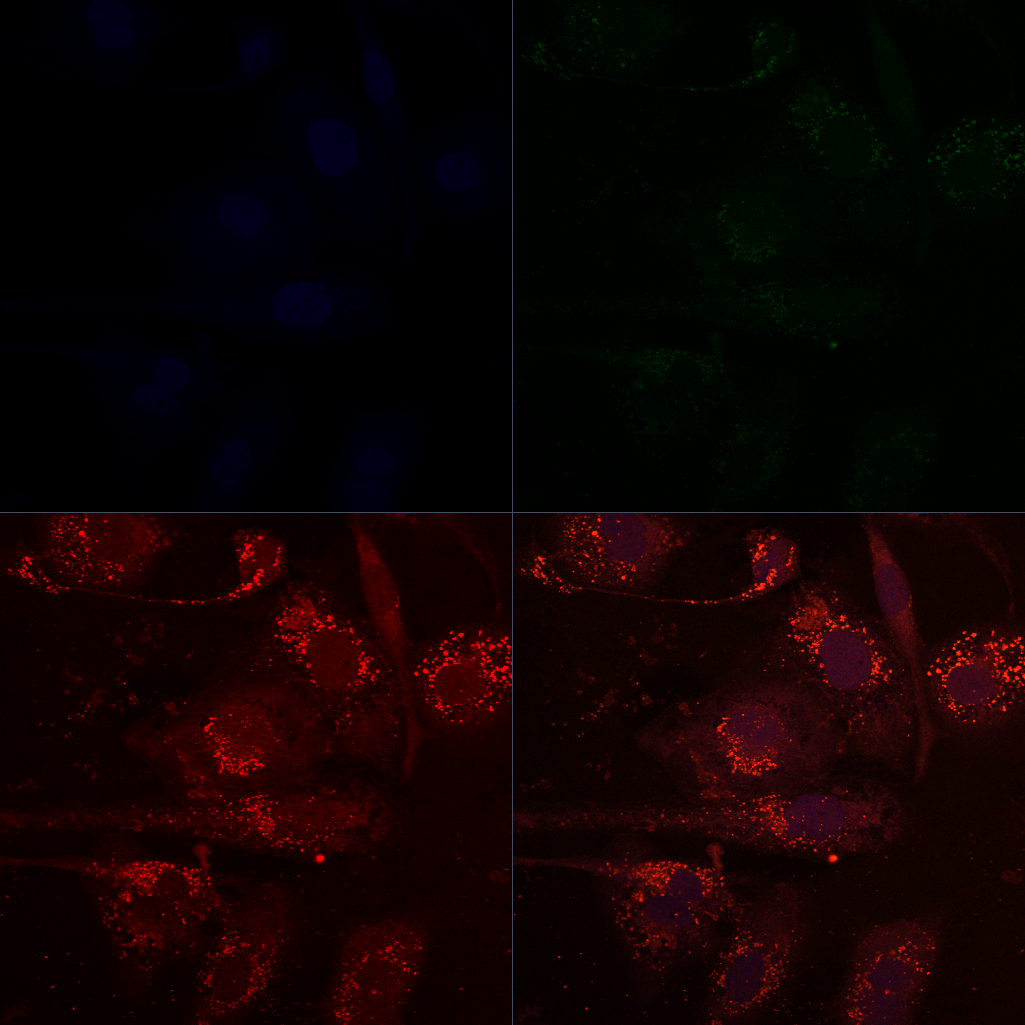

Supplement: S5 Fig — (TIF) [file pone.0151767.s005.tif]

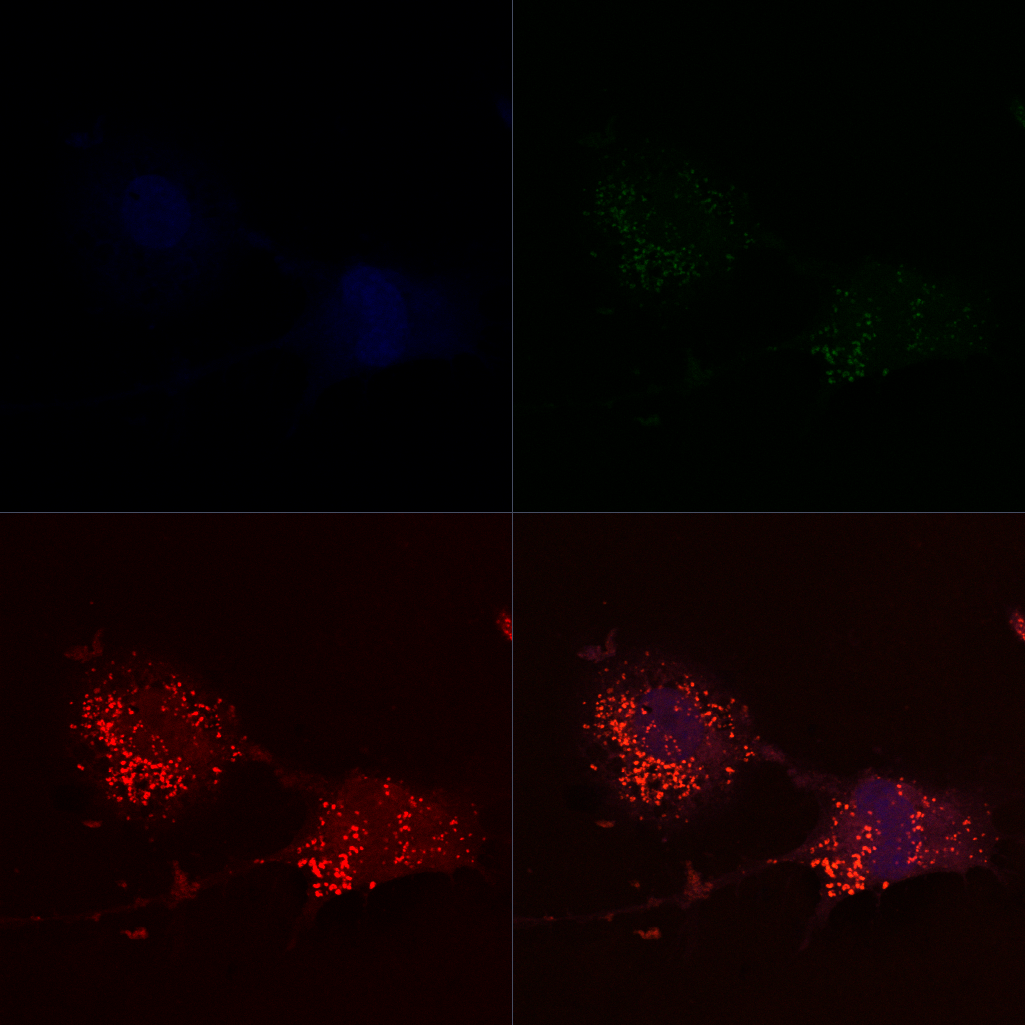

Supplement: S6 Fig — (TIF) [file pone.0151767.s006.tif]

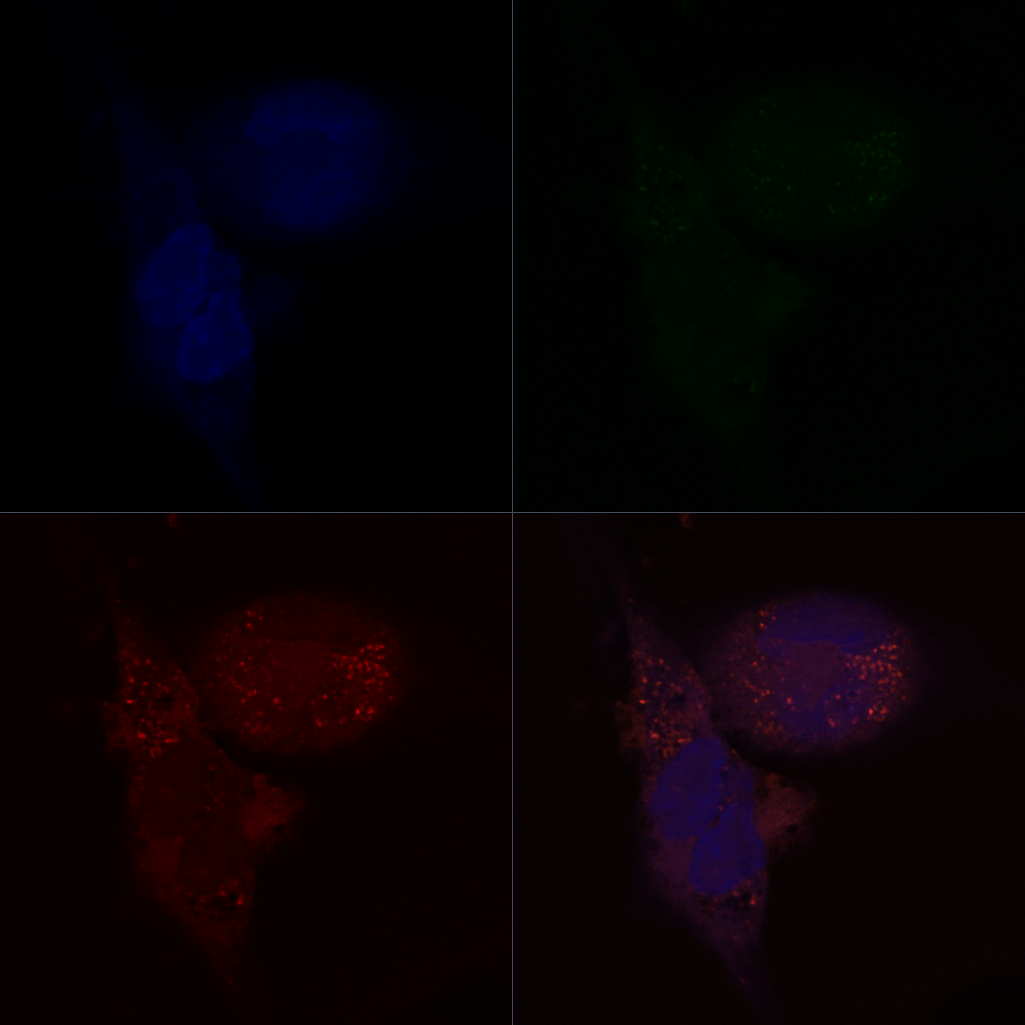

Supplement: S7 Fig — This indicates that apoA-1 promotes cholesterol efflux from the macrophages. (TIF) [file pone.0151767.s007.tif]

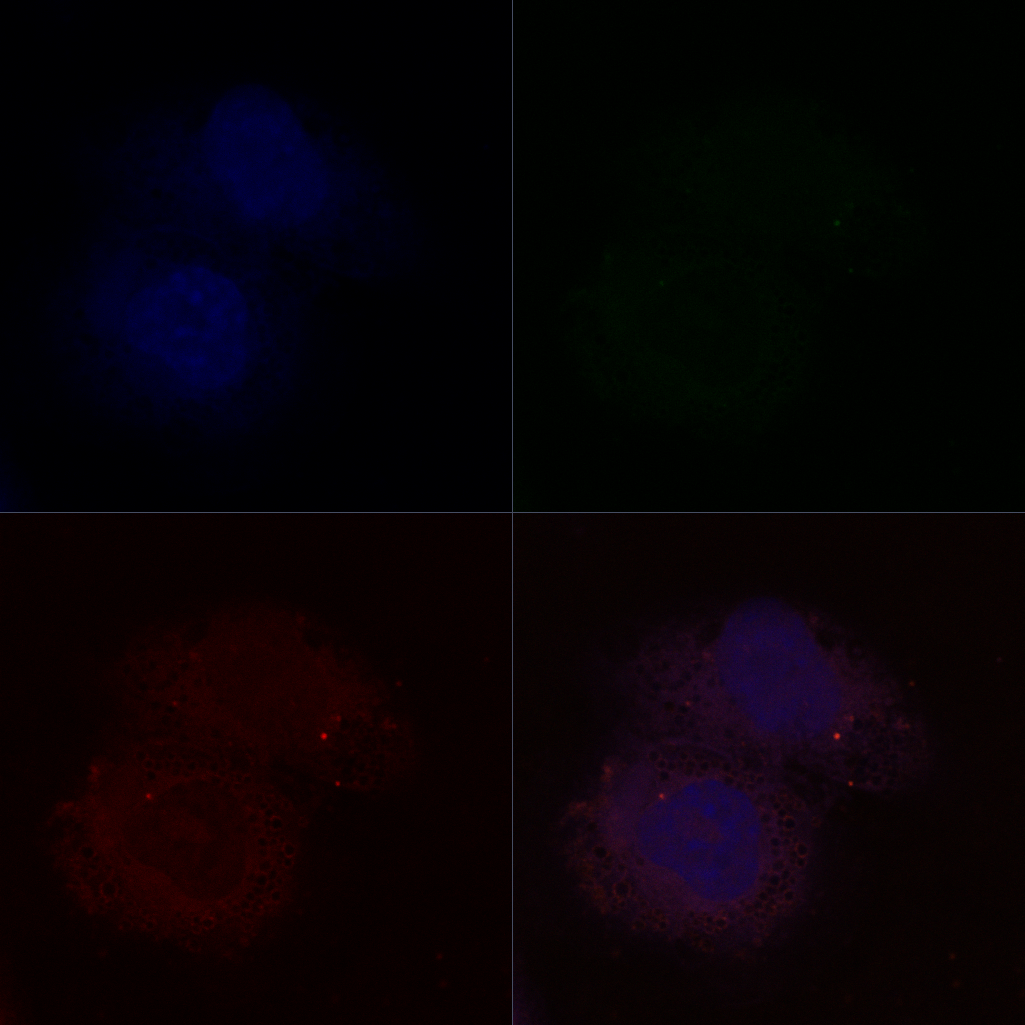

Supplement: S8 Fig — (TIF) [file pone.0151767.s008.tif]

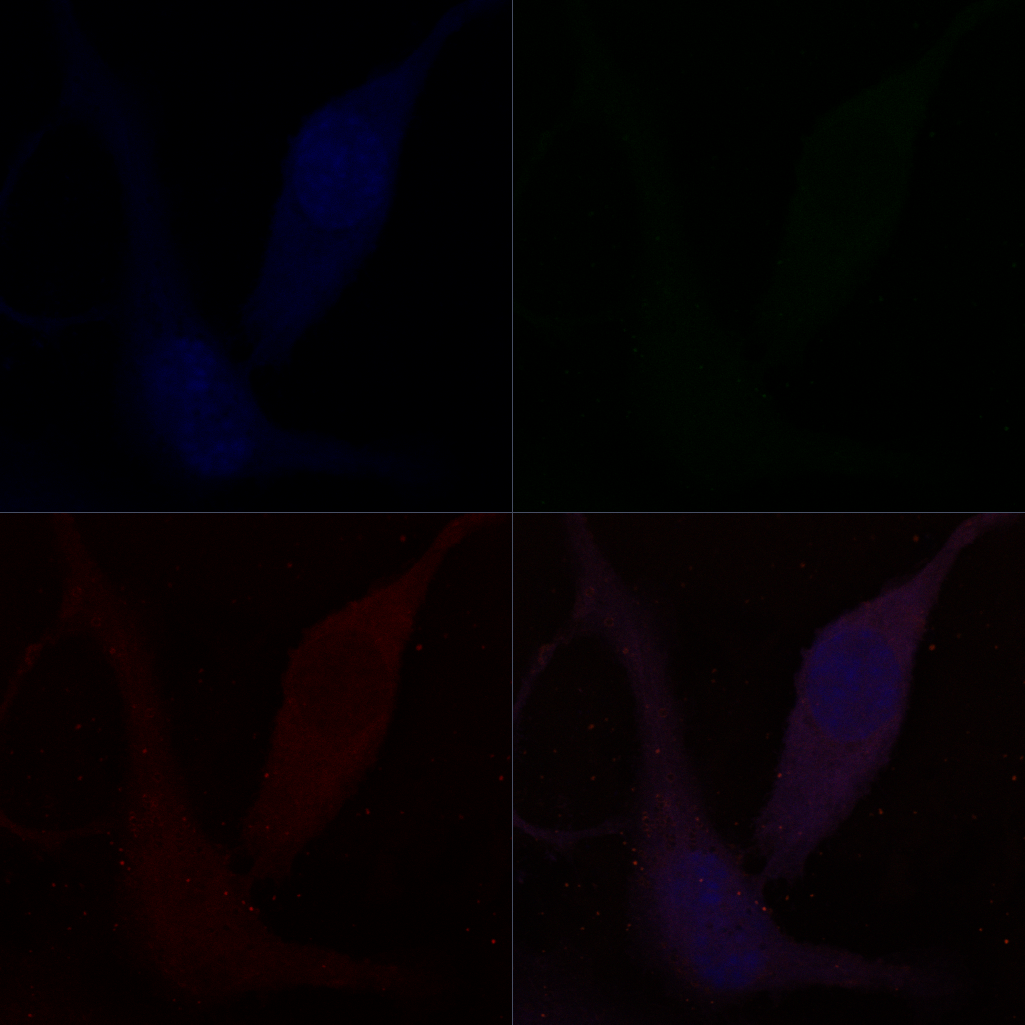

Supplement: S9 Fig — (TIF) [file pone.0151767.s009.tif]

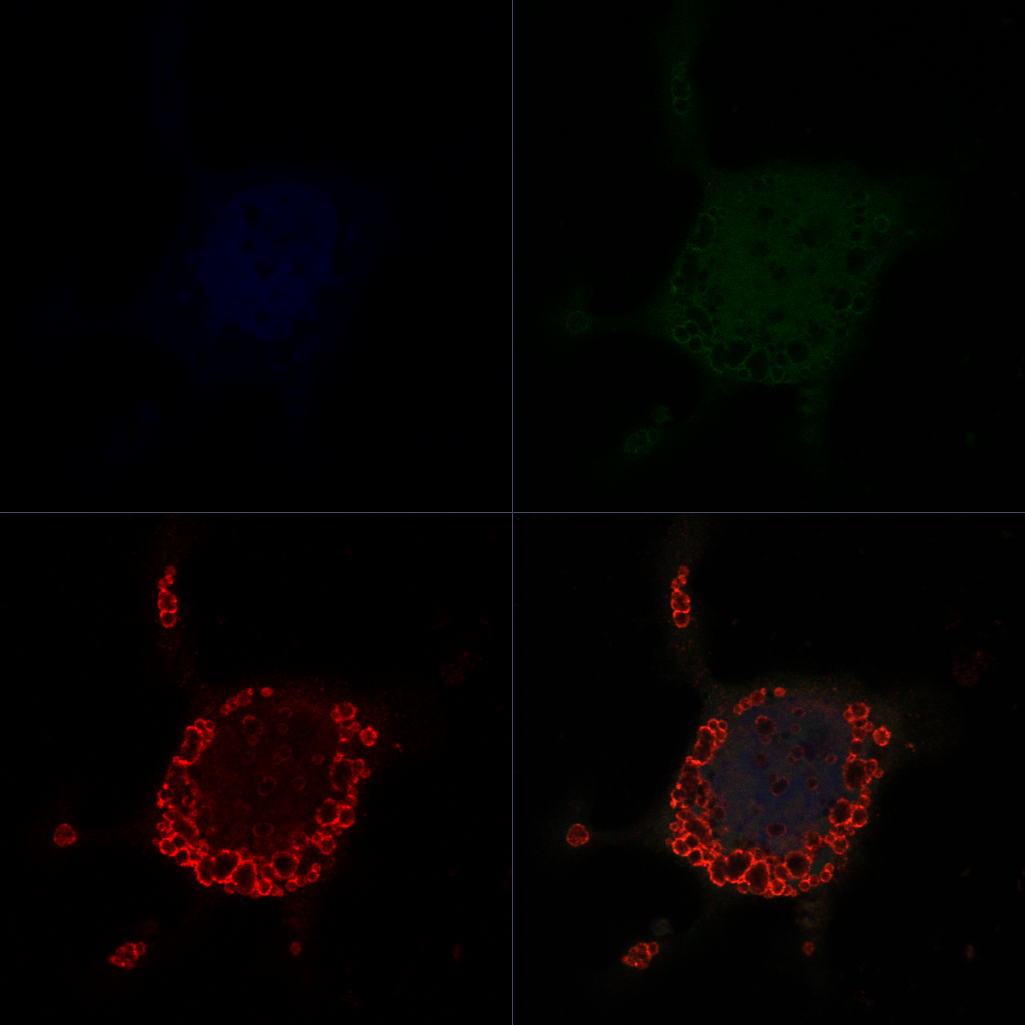

Supplement: S10 Fig — Vesicles with intense fluorescence are clearly visible and some vesicles are arranged in beautiful rosettes, supporting the concept that reduction of β-COP prevents cholesterol removal from the cells by apoA-1 mediated exocytotic pathway and leads to the cholesterol accumulation in the vesicles. The green channel also shows increased expression of the GFP from the β-COP shRNA lentivirus. (TIF) [file pone.0151767.s010.tif]

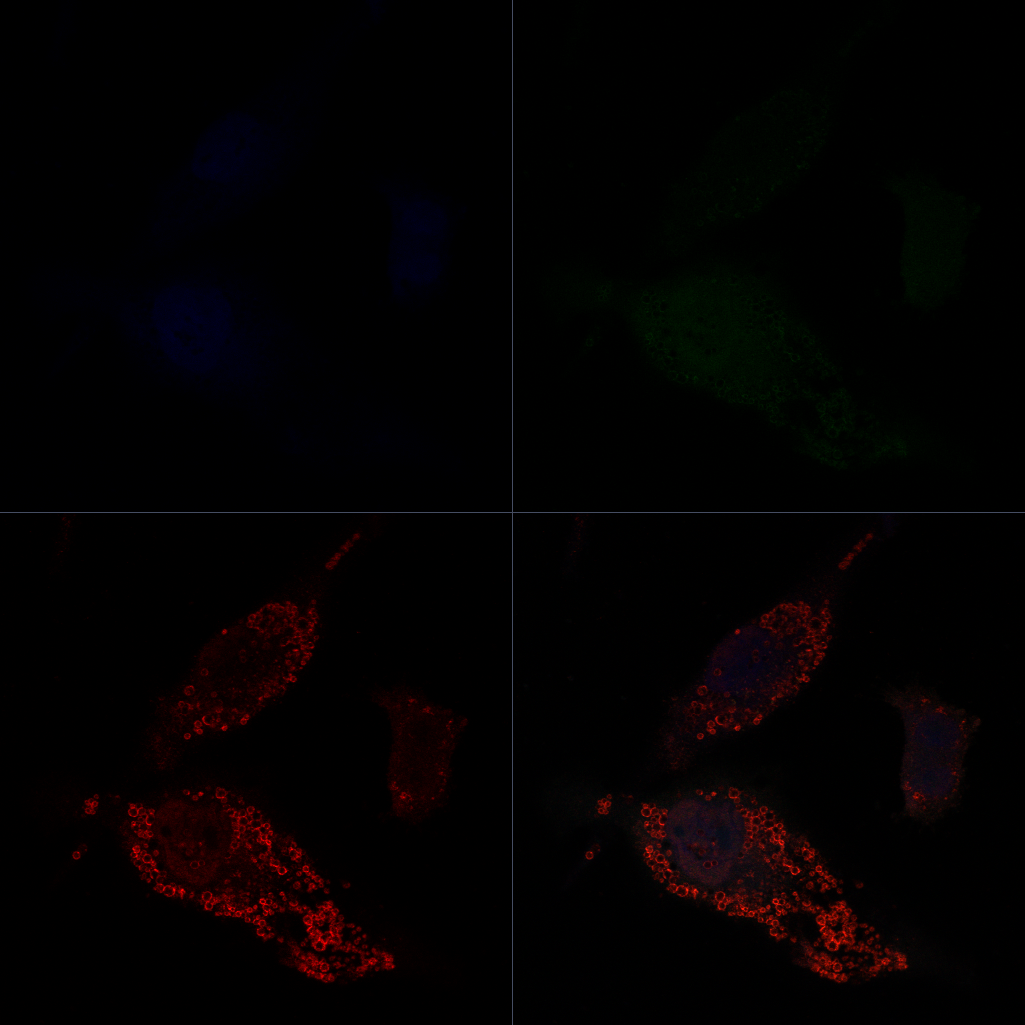

Supplement: S11 Fig — Vesicles with intense fluorescence are clearly visible as the S10 Fig. (TIF) [file pone.0151767.s011.tif]

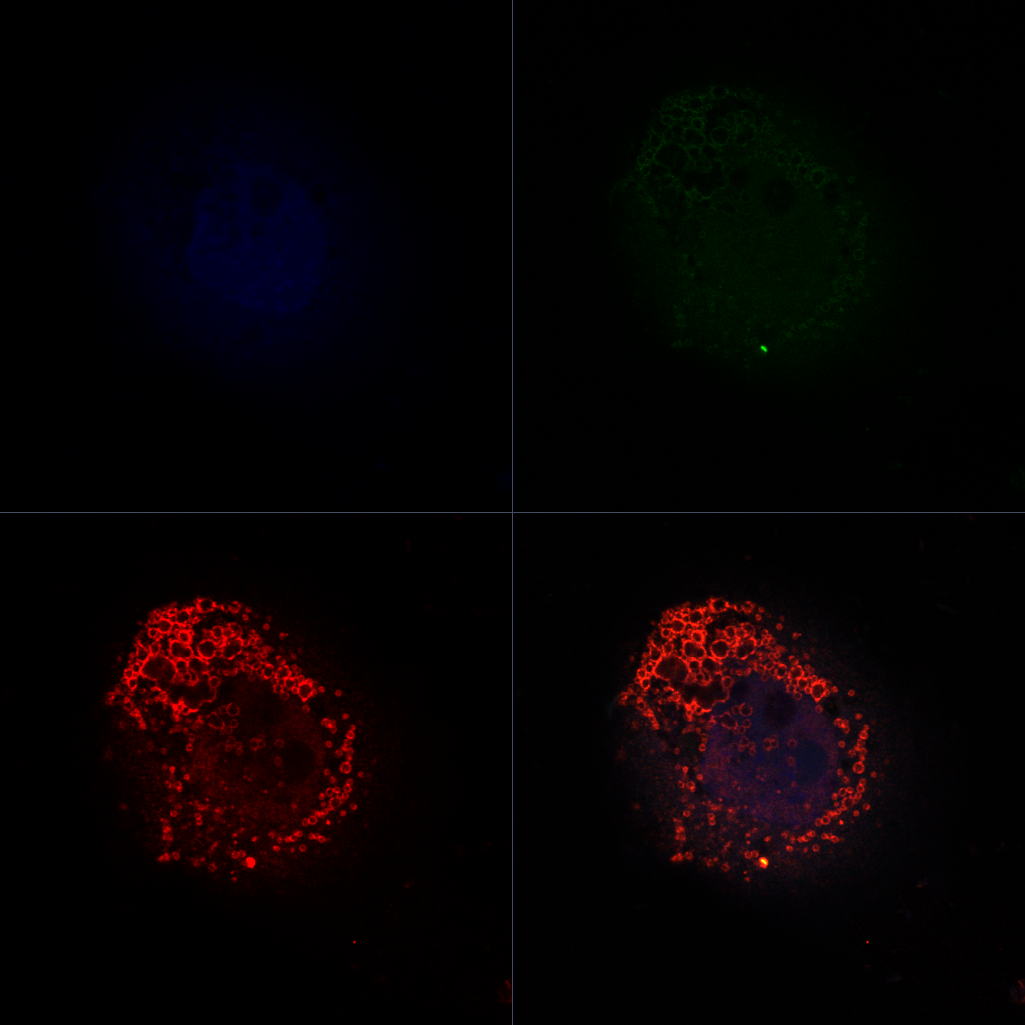

Supplement: S12 Fig — Vesicles with intense fluorescence are clearly visible as the S10 and S11 Figs. (TIF) [file pone.0151767.s012.tif]

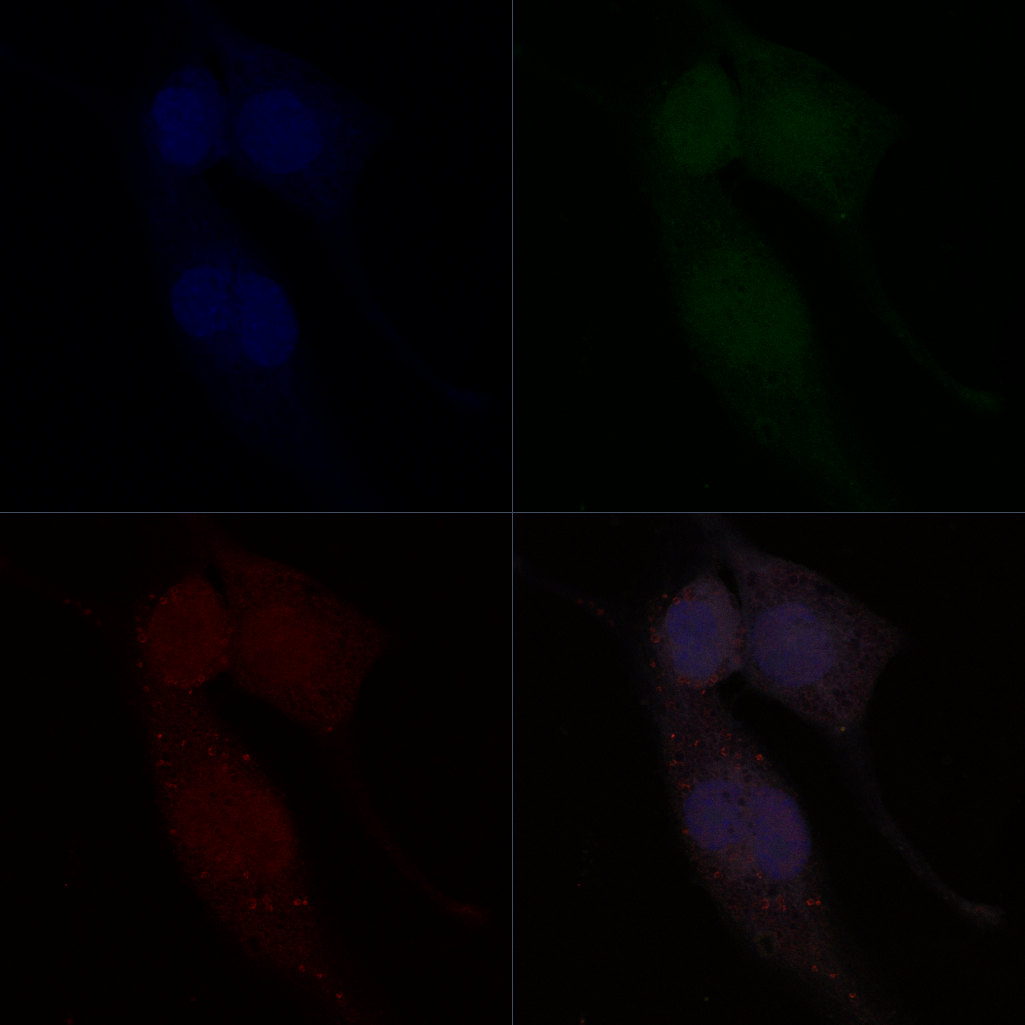

Supplement: S13 Fig — Red fluorescence is very low and the green fluorescence is visible, indicating that active expression of scrambled shRNA takes place in these cells but has minimal effect on apoA-1 mediated cholesterol efflux from the macrophages. (TIF) [file pone.0151767.s013.tif]

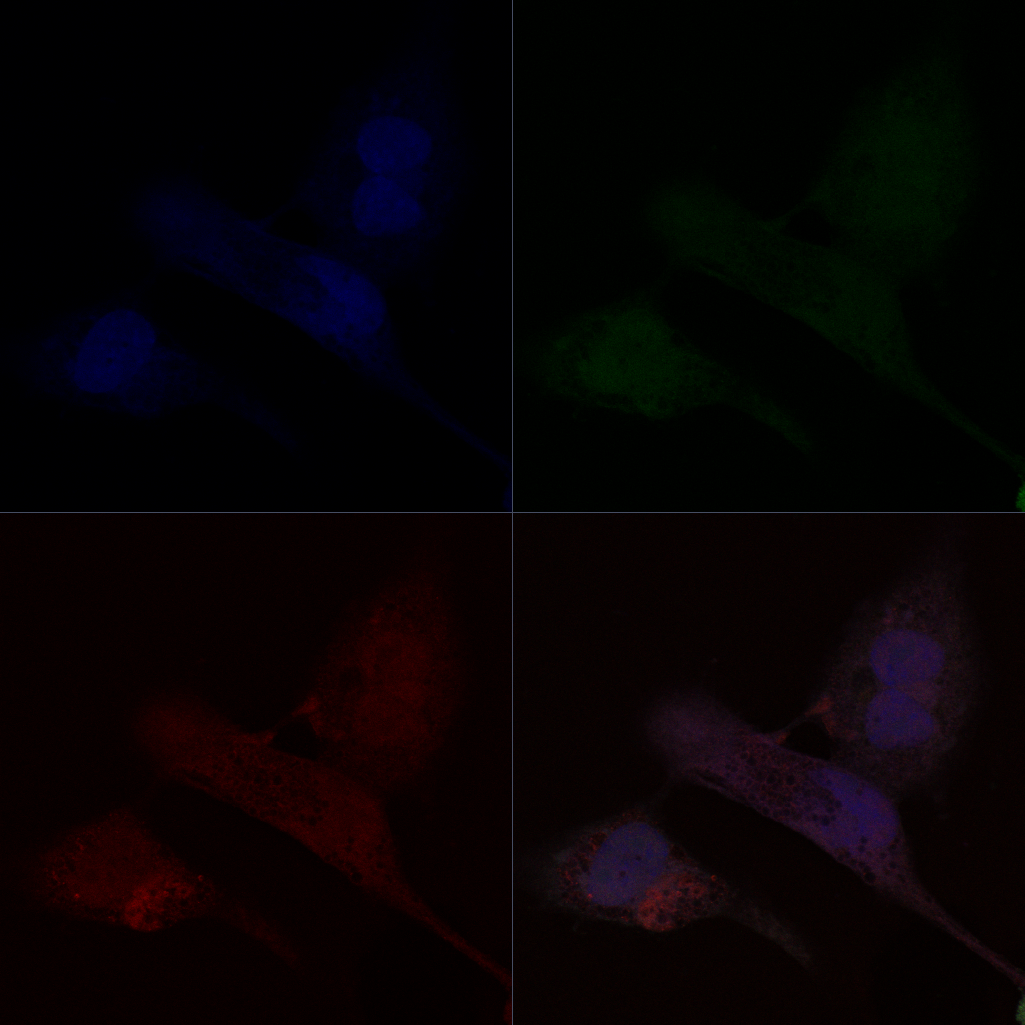

Supplement: S14 Fig — The cells have low red fluorescence as the S13 Fig. (TIF) [file pone.0151767.s014.tif]

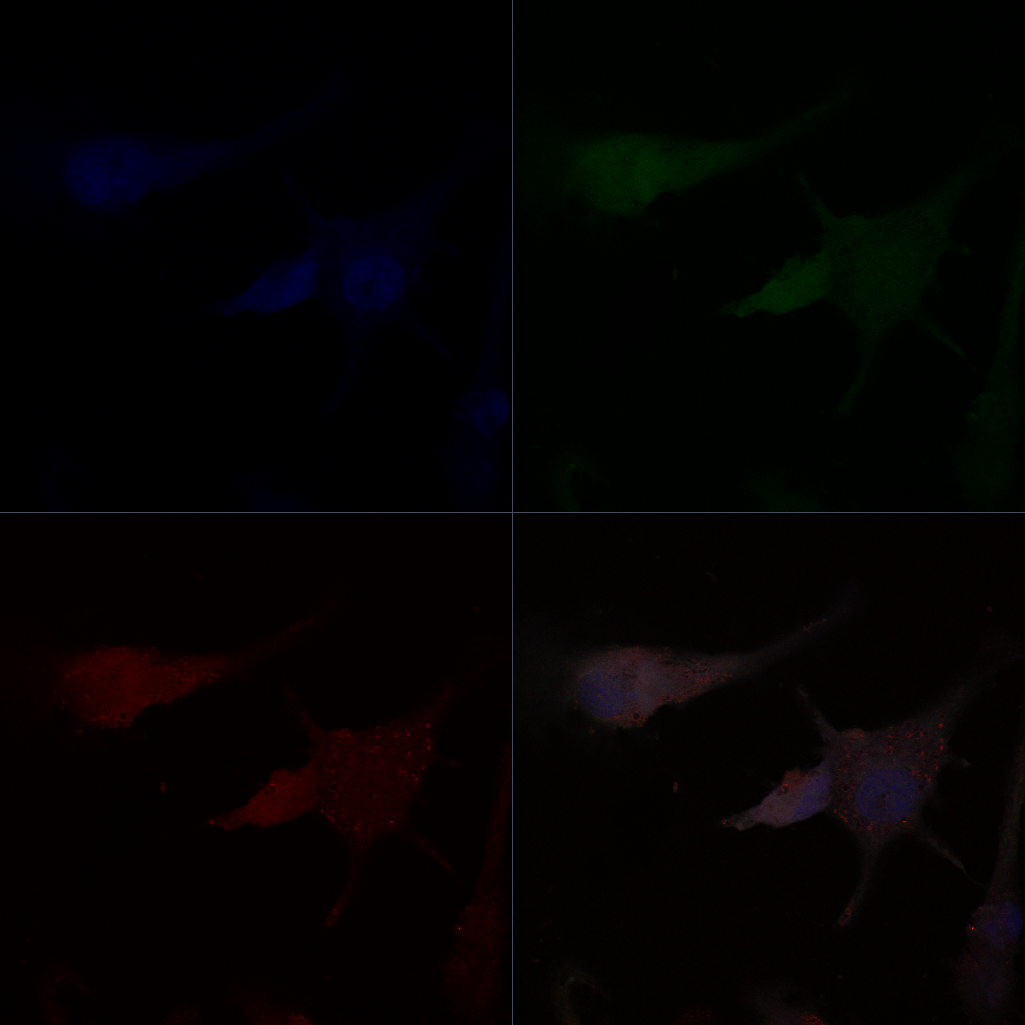

Supplement: S15 Fig — The cells have low red fluorescence as the S13 Fig and Fig 14. (TIF) [file pone.0151767.s015.tif]
